# Cdk8/CDK19 promotes mitochondrial fission through Drp1 phosphorylation and can phenotypically suppress *pink1* deficiency in Drosophila

**DOI:** 10.1038/s41467-024-47623-8

**Published:** 2024-04-18

**Authors:** Jenny Zhe Liao, Hyung-lok Chung, Claire Shih, Kenneth Kin Lam Wong, Debdeep Dutta, Zelha Nil, Catherine Grace Burns, Oguz Kanca, Ye-Jin Park, Zhongyuan Zuo, Paul C. Marcogliese, Katherine Sew, Hugo J. Bellen, Esther M. Verheyen

**Affiliations:** 1 Department of Molecular Biology and Biochemistry, Simon Fraser University, Burnaby, V5A1S6 BC Canada; 2 Center for Cell Biology, Development and Disease, Simon Fraser University, Burnaby, V5A1S6 BC Canada; 3 Department of Neurology, Houston Methodist Research Institute, Houston, TX USA; 4 Department of Molecular and Human Genetics, Jan and Dan Duncan Neurological Institute, Baylor College of Medicine, Houston, TX 77030 USA; 5 Department of Biochemistry and Medical Genetics, University of Manitoba, Winnipeg, R3E0J9 MB Canada; 6 Children’s Hospital Research Institute of Manitoba, Winnipeg, R3E3P4 MB Canada; 7 Department of Neuroscience, Baylor College of Medicine, Houston, TX 77030 USA; 8 Present Address: Department of Biology, Stanford University, Stanford, CA 94305 USA

**Keywords:** Mitochondria, Cellular neuroscience, Ageing, Kinases

## Abstract

*Cdk8* in Drosophila is the orthologue of vertebrate *CDK8* and *CDK19*. These proteins have been shown to modulate transcriptional control by RNA polymerase II. We found that neuronal loss of *Cdk8* severely reduces fly lifespan and causes bang sensitivity. Remarkably, these defects can be rescued by expression of human CDK19, found in the cytoplasm of neurons, suggesting a non-nuclear function of CDK19/Cdk8. Here we show that Cdk8 plays a critical role in the cytoplasm, with its loss causing elongated mitochondria in both muscles and neurons. We find that endogenous GFP-tagged Cdk8 can be found in both the cytoplasm and nucleus. We show that Cdk8 promotes the phosphorylation of Drp1 at S616, a protein required for mitochondrial fission. Interestingly, Pink1, a mitochondrial kinase implicated in Parkinson’s disease, also phosphorylates Drp1 at the same residue. Indeed, overexpression of Cdk8 significantly suppresses the phenotypes observed in flies with low levels of Pink1, including elevated levels of ROS, mitochondrial dysmorphology, and behavioral defects. In summary, we propose that Pink1 and Cdk8 perform similar functions to promote Drp1-mediated fission.

## Abstract (web summary)

Mitochondrial fission, performed by Drp1, is carefully regulated, particularly in neurons. Here, the authors examine Drosophila Cdk8/CDK19 function in mitochondrial fission and uncover a role phosphorylating Drp1 in the cytoplasm and show overexpression suppresses a Parkinson’s disease model.

## Introduction

Cyclin-dependent kinase 19 (CDK19 [MIM: 614720]) and its paralog, CDK8 (MIM: 603184), are members of the transcriptional family of CDKs. Unlike other CDKs, these CDKs are not directly involved in cell-cycle regulatory processes^1^. Both CDK19 and CDK8 interact with Cyclin C to form a conserved kinase module that regulates RNA polymerase II-mediated transcription in the Mediator protein complex^2^. Enhanced CDK8 activity is implicated in tumorigenesis, including in colorectal cancer, breast cancer, and melanoma^3–6^.

We and others recently showed that mutations in *CDK8/CDK19* are associated with severe neurodevelopmental disorders in humans^7–10^. Most patients with CDK19 pathogenic variants exhibit epilepsy, including infantile spasms, hypotonia, ataxia, developmental delay/severe intellectual disability, central nervous system (CNS) changes on MRI (brain atrophy, delayed myelination), and craniofacial dysmorphisms. Flies that lack *cdk8* in neurons, the orthologue of *CDK8/CDK19*, exhibit similar phenotypes such as reduced lifespan and susceptibility to seizures^9^. Most de novo variants identified in *CDK8* and *CDK19* map to the ATP-binding domain associated with the kinase activity^7–10^. Interestingly, CDK19 tagged with HA, when expressed in flies, rescued defects associated with the loss of *cdk8* in neurons, demonstrating functional conservation. Strikingly, the exogenous CDK19 protein was localized primarily in the cytoplasm of neurons, not the nucleus, suggesting that Cdk8/CDK19 may play an important role in the cytoplasm.

In this study, we show that depletion of *Cdk8* in muscle and neurons causes a loss of mitochondrial fission, a phenotype that is also observed when Drp1, a protein required for mitochondrial fission, is lost^11^. These defects can be rescued by the expression of a cytoplasmically targeted CDK19 protein, indicating a non-nuclear role for Cdk8 family members. We endogenously tagged Cdk8 and found a dynamic subcellular distribution, including both cytoplasmic and nuclear localization in diverse tissues. We show that Cdk8 binds to Drp1, increases the phosphorylation of Drp1 at S616, and that loss of Cdk8 decreases the phosphorylation of Drp1 S616. Loss of Pink1, a kinase required for phosphorylation of Drp1 at S616, causes defects in mitochondrial dynamics, mitophagy, and Complex I function. Mutations in *PINK1* and *PARKIN* have been identified in patients with juvenile Parkinsonism, a familial form of Parkinson’s Disease (PD)^12–15^. Pink1 is a serine/threonine kinase that regulates mitochondrial quality control^16–19^. *pink1* mutant flies exhibit phenotypes resembling symptoms of PD patients, including defects in locomotor activity, reduced lifespan, and accumulation of dysfunctional mitochondria^16,17,20–23^. Overexpression of Cdk8 and both wildtype and cytoplasmic CDK19 rescues phenotypes associated with loss of *pink1*. In summary, our data reveal a new function for Cdk8/CDK19 in mitochondrial fission by mediating the phosphorylation of Drp1 and this function parallels the function of *pink1*.

## Results

### Depletion of *Cdk8* causes altered wing posture, reduced lifespan, and defects in locomotion

**Fig. 1:**
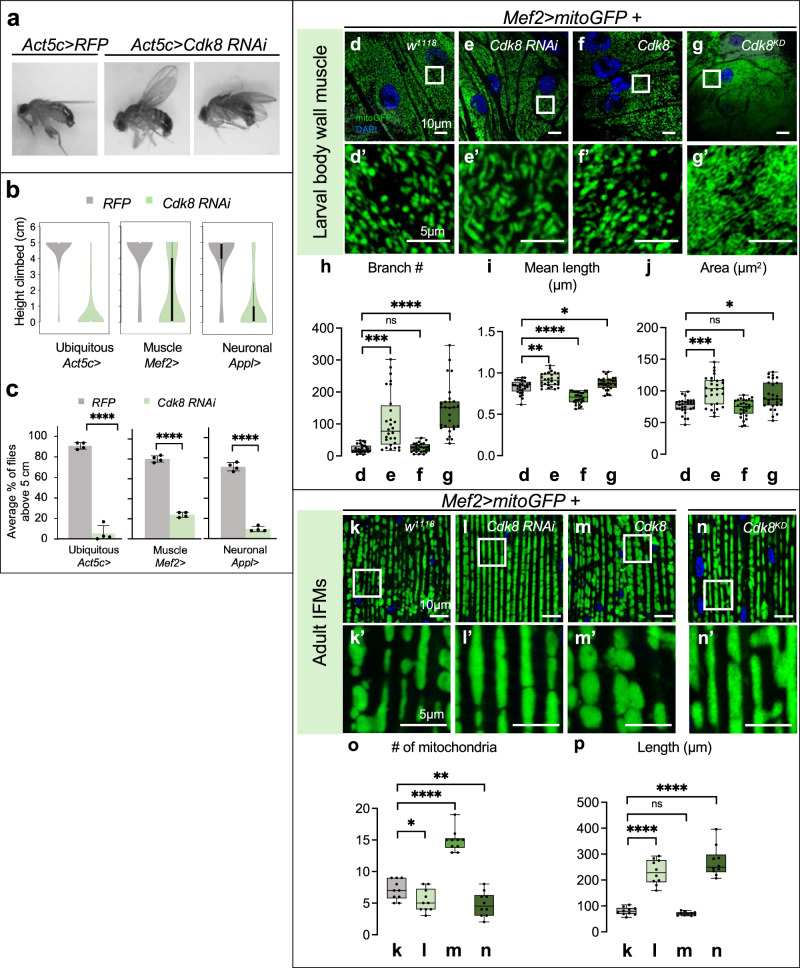
Cdk8 regulates mitochondrial morphology under physiological conditions. **a** Wing postures of flies expressing *Act5c*>*RFP* and *Act5c>Cdk8* RNAi. **b** Violin plots showing distributions of climbing ability in control (RFP) and *Cdk8* knockdown flies using either ubiquitous (Act5c), muscle (Mef2), or neuronal-specific (Appl) Gal4 drivers. **c** Average percent of flies that reached the target line of indicated genotypes in a climbing assay. Each experiment is individually repeated four times. The total number of progeny are assessed between *white RNAi* and *Cdk8 RNAi* with different drivers: *n* = 165, 144 in *Act5*>, 180, 189 in *Mef2*>, and 160, 160 in *App*>, respectively. Results are mean ± SD. An unpaired two-tailed *t*-test is used to generate the statistics. *P* value is 8.71e-07 for *Act5c-Gal4*, 1.67e-07 for *Mef2-Gal4*, and 2.32e-07 for *Appl-Gal4*. All raised at 25 °C. **d**–**g** Mitochondrial morphology of larval body wall muscles in (**d**) *w*^*1118*^ control or expressing (**e**) *Cdk8 RNAi*, (**f**) *Cdk8*^*WT*^, (**g**) *Cdk8*^*KD*^ with *Mef2-Gal4>mitoGFP*, raised at 29 °C. Scale bar: 1 μm. **d**’–**g**’ Representative magnified sections from (**d**–**g**). Scale bar: 5 μm. **h**–**j** Quantification of mitochondrial morphology showing the (**h**) number of branches. *P* value is 0.0003 for *Cdk8 RNAi*, 0.4533 for *Cdk8*, and 6.97e-10 for *Cdk8*^*KD*^ (**i**) the mean mitochondrial length. *P* value is 0.0023, 6.29e-8, and 0.0178, respectively, in order. **j** The mitochondrial area. *P* value is 0.0002, 0.5512, and 0.0167, respectively, in order, in larval body wall muscles of the indicated genotypes. All in comparison to *w1118*. Letters (**d**–**g**) refer to the genotypes shown in panels (**d**–**g**). Quantifications were calculated by the MiNA toolset. *n* = 6 per genotype. Data were presented as box plots (center line at the median, upper bound at 75^th^ percentile, lower bound at 25th percentile) with whiskers at minimum and maximum values. An unpaired two-tailed *t*-test is used to generate the statistics. **k**–**n** Mitochondrial morphology of adult IFM in (**k**) *w*^*1118*^ control or expressing (**l**) *Cdk8 RNAi*, (**m**) *Cdk8*^*WT*^, (**n**) *Cdk8*^*KD*^ with *Mef2-Gal4>mitoGFP*. Flies were grown at 29 °C, except escapers expressing Cdk8^KD^ were raised at 25 °C. Scale bar: 10 μm. **k**’–**n**’ Representative magnified sections from (**k**–**n**). Scale bar: 5 μm. **o**–**p** Quantification of mitochondrial morphology showing the (**h**) number of mitochondria, *p* value is 0.0371 for *Cdk8 RNAi*, 4.25e-9 for *Cdk8*, and 0.0095 for *Cdk8*^*KD*^. **i** The mean mitochondrial length of the indicated genotypes. *P* value is 1.56e-8, 0.l0809, and 7.89e-9, respectively in order. All in comparison to *w1118*. *n* = 5 per genotype. Data were presented as box plots (center line at the median, upper bound at 75^th^ percentile, lower bound at 25th percentile) with whiskers at minimum and maximum values. An unpaired two-tailed *t*-test is used to generate the statistics. ∗*p* < 0.05, ∗∗*p* < 0.01, ∗∗∗*p* < 0.001, ∗∗∗∗*p* < 0.0001, ns no significance. Source data are provided as a Source Data file.

To investigate the consequences of reduced Cdk8 levels in flies, we examined the effects of ubiquitous knockdown of *Cdk8* using the *Act5c-Gal4* strain (*Act5c>Cdk8 RNAi). Cdk8* expression was reduced by ~80% (Fig. S1a), and only about 50% of the flies eclosed at 25 °C (Fig. S1b). The flies that eclosed exhibit abnormal wing postures with either held-up or droopy wings shortly after eclosion (Fig. 1a). They also have significantly shorter life spans (Fig. S1c) and are sterile (Fig. S1d). We observed that *Act5c>Cdk8 RNAi* have impaired climbing abilities (Fig. 1b, c). Knocking down *Cdk8* using either a muscle-specific (*Mef2-Gal4*) or a neuron-specific (*Appl-Gal4*) driver caused similar, though slightly less severe, climbing defects (Fig. 1b, c) when compared to ubiquitous knockdown. When a kinase-dead version of Cdk8 (*Cdk8*^*KD*^), which carries a single amino acid mutation within the well-conserved kinase domain (T173D, ref. ^24^), was expressed in muscles using *Mef2-Gal4* it caused similar reduced survival (Fig. S1e) and severe climbing defects in escaper adults (Fig. S1f).

These phenotypes mimic phenotypes seen in flies with wing hinges and muscle defects^25,26^, including flies in which *pink1* or *parkin* are depleted^16,17,21,27^. Mutations in *PINK1* cause a familial form of Parkinson’s Disease^27–29^ and loss of *pink1* disrupts mitochondrial homeostasis and impairs mitophagy^23^. Since the Cdk8 module is a well-known transcriptional regulator, we asked if the phenotypes were due to its role in regulating the transcription of *pink1* and *parkin* using qRT-PCR. We found no significant differences in mRNA levels of *pink1* or *parkin* when either *Cdk8* or *CycC* were knocked down ubiquitously (Fig. S2a, b).

### Cdk8 regulates mitochondrial morphology

Our previous data suggest that Cdk8/CDK19 may have a critical function in the cytoplasm. Given that most patients with de novo variants in *CDK19* exhibit muscle hypotonia^9^ and that numerous genetic diseases that affect mitochondrial function cause hypotonia^30^, we decided to explore mitochondrial phenotypes in muscles. We further investigated the mitochondrial status following *Cdk8* depletion, given the phenotypic similarity (Fig. 1a) to *pink1* mutants that exhibit muscle defects. Mitochondria are highly dynamic and undergo fission and fusion^31,32^. We examined mitochondrial morphology in larval muscles using a muscle-specific driver and labeled mitochondria with mitoGFP^33^. In control larval body wall muscles (*Mef2>mitoGFP*/+), we observe punctate and filamentous structures (Fig. 1d). However, when *Cdk8* levels are reduced using RNAi, we observe an increase in branch length, mitochondrial length, and total mitochondrial area (Fig. 1e, e’, h–j and Fig. S3b, c’). In contrast, overexpression of the wild-type *Cdk8* cDNA does not affect branch number but causes a decrease in length (Fig. 1f, f’, h–j and Fig. S3d, e’). Moreover, overexpression of a kinase-dead version of Cdk8 (*Cdk8*^*KD*^) leads to very elongated mitochondria with significantly increased numbers of branches (Fig. 1g, g’ and Fig. S3f, f’). In summary, loss of *Cdk8* or expression of kinase-dead Cdk8 affects mitochondrial dynamics in developing muscles.

We next examined mitochondrial morphology in adult indirect flight muscles (IFMs). In control animals, we observe both elongated and fragmented mitochondria (Fig. 1k, k’). *Cdk8* depletion causes elongated mitochondria (Fig. 1l, l’), whereas overexpression of Cdk8 leads to small and round mitochondria, indicative of increased fission (Fig. 1m, m’). Since expression of Cdk8^KD^ causes pupal lethality at 29 °C (Fig. S1e), we examined the mitochondrial morphology of escapers raised at 25 °C (Fig. 1n, n’) and again observed elongated mitochondria. These results were similarly observed when we tested the mitochondrial morphology using a different RNAi line for Cdk8 (Fig. S3b, c) or *UAS-Cdk8-HA* (Fig. S3i, j’). Since the Cdk8 module can modulate gene expression, we tested the effect of Cdk8 and Cyc C on the transcription of regulators of mitochondrial dynamics. Ubiquitous knockdown of either *Cdk8* or *Cyc C* led to a modest increase in expression of both a regulator of mitochondrial fusion (mitochondrial assembly regulatory factor, *marf*) and fission (*Drp1*), suggesting the mitochondrial morphology defects are not likely due to reduced mRNA levels of these genes (Fig. S3n). Cyclin C (Cyc C) has previously been shown to exit the nucleus under oxidative stress conditions to regulate mitochondrial morphology in yeast and mouse embryonic fibroblast^34–36^. Depletion of *Cyc C* in muscle leads to elongation of mitochondria, and overexpression of Cyc C promotes fragmentation of mitochondria (Fig. S3g, h’). In summary, both Cdk8 and Cyc C modulate mitochondrial morphology, and proper expression of Cdk8 and Cyc C maintains the proper balance in mitochondrial dynamics. To test whether Cdk8 might function by promoting the activity of Cyc C, we tested whether Cyc C expression could rescue Cdk8 depletion phenotypes (Fig. S2c). While Cdk8 expression rescued the mitochondrial phenotypes caused by an expression of *Cdk8-RNAi*, Cyc C expression failed to rescue the phenotypes. We also performed the reciprocal experiment in which we tested whether Cdk8 could rescue the effects of *Cyc C* knockdown (Fig. S2d). Cdk8 expression alters mitochondrial abnormalities caused by an expression of *Cyc C-RNAi* but does not rescue. However, expression of Cyc C can rescue the fusion caused by the depletion of Cyc C. Hence, these results suggest that Cdk8 and Cyc C use distinct mechanisms to modulate mitochondrial dynamics.

### Loss of Cdk8 alters mitochondrial distribution in the CNS neuropil

**Fig. 2:**
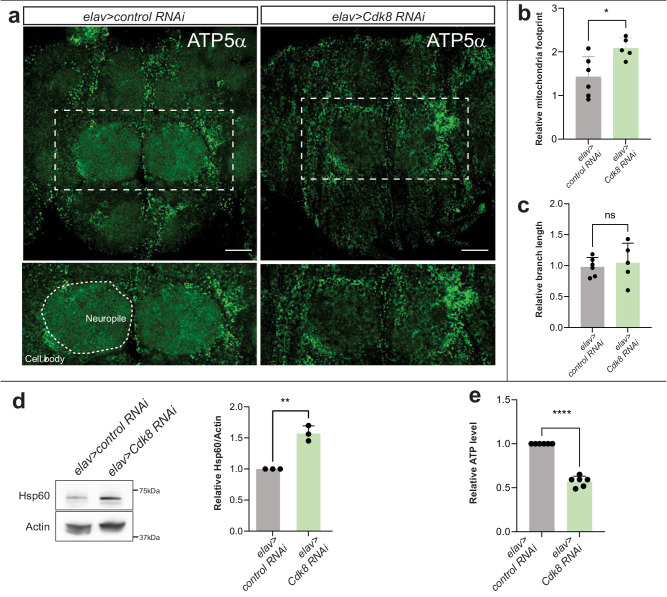
Cdk8 is required in the mitochondrial distribution in some neurons of adult CNS. **a** Mitochondrial distribution in the CNS neuropil. ATP5a marks mitochondria in the brain of control (*elav>control RNAi*) and flies that express *Cdk8 RNAi* (*elav>Cdk8 RNAi*). Scale bar: 10 μm. **b** Quantification of relative mitochondrial footprint (*n* = 6 for control, *n* = 5 for *elav>Cdk8 RNAi*). **c** Quantification of relative mitochondrial branch length (*n* = 6 for control, *n* = 5 for *elav>Cdk8 RNAi*). **d** Western blot and quantification of Hsp60 from the heads of control (*elav>control RNAi*) and flies that express *Cdk8 RNAi* (*elav>Cdk8 RNAi*) (*n* = 3 per genotype). *p* = 0.0013. **e** Quantification of ATP level in the heads of control (*elav>control RNAi*) and flies that express *Cdk8 RNAi* (*elav>Cdk8 RNAi*) (*n* = 6 per genotype). Statistical analyses are one-way ANOVA followed by a Tukey post hoc test. Results are mean ± SEM (∗*p* < 0.05, ∗∗*p* < 0.01, ∗∗∗∗*p* < 0.0001, ns no significance). Source data are provided as a Source Data file.

Given that patients with *CDK19* variants develop severe defects in neurodevelopment, we next assessed the effects of depletion of *Cdk8* in the nervous system. To assess mitochondrial distribution in the adult CNS, we expressed *Cdk8 RNAi* in neurons using a pan-neuronal driver, *elav-Gal4*, and stained the mitochondria with an anti-ATP5 antibody. As shown in Fig. 2a, mitochondria are indeed more clustered in cell bodies, and less abundant in the neuropil when compared to control animals (*elav>control RNAi*) (Fig. 2a). We further quantified the length of mitochondrial area and branches to assess the morphological changes. As shown in Fig. 2b, the area of mitochondria is significantly increased, but branch length is not altered (Fig. 2c).

As the loss of mitochondrial fission can lead to mitochondrial stress^37^ we quantified mitochondrial stress by measuring the level of the mitochondrial stress protein Hsp60^38^. As shown in Fig. 2d, mitochondrial stress is significantly increased in adult brains. Furthermore, the function of mitochondria is also affected as the ATP synthesis level is significantly reduced upon depletion of *Cdk8* (Fig. 2e). Taken together, our data indicate that loss of *Cdk8* affects mitochondrial dynamics, which results in a mitochondrial transport defect into the neuropil as well as a dysfunction of the mitochondria.

Abnormal mitochondrial morphology can elevate the production of reactive oxygen species (ROS). Therefore, we performed dihydroethidium (DHE) staining to assess ROS in adult brains. Adult brains of *elav>cdk8 RNAi* shows a 40% higher level of ROS when compared to control animals (*elav>Luciferase RNAi*) (Fig. S4a, b). Flies expressing human CDK19 in a *Cdk8*-depleted background decrease the level of ROS by 20% (Fig. S4a, b), showing that CDK19 can partially rescue the elevated ROS due to loss of *Cdk8*. Similarly, expression of *Cdk8 RNAi* in adult indirect flight muscles (*Mef2>Cdk8 RNAi*) significantly increased the level of ROS when compared to the control muscles (*Mef2>white RNAi*), however overexpression of fly Cdk8 does not significantly affect the level of ROS (Fig. S4c–g).

### Loss of *Cdk8* in photoreceptors affects mitochondrial size and distribution in photoreceptor terminals

**Fig. 3:**
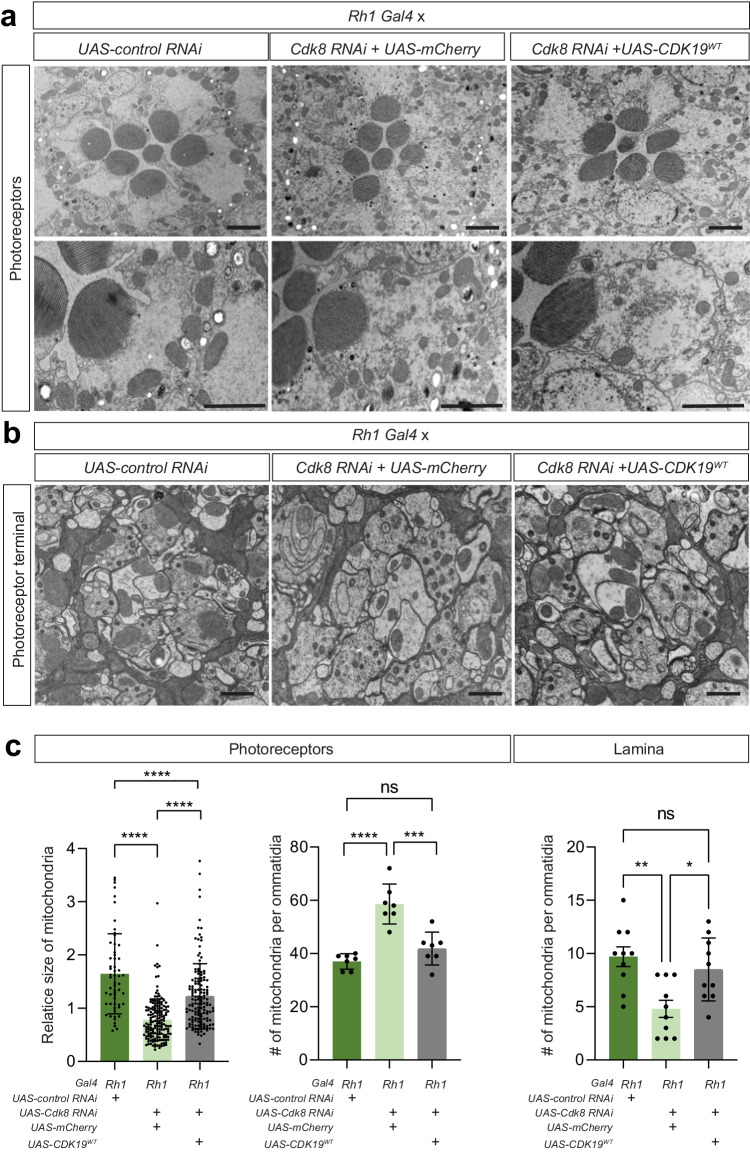
Loss of Cdk8 affects mitochondrial size and distribution in photoreceptor terminals, which can be rescued by CDK19. **a** Mitochondrial distribution in the cell bodies of the photoreceptors of the indicated genotypes. Scale bars: 2 μm. **b** TEM images of photoreceptor terminals. The size of mitochondria is smaller than in the control but observed more mitochondria in clusters in photoreceptors of flies that express *Cdk8 RNAi* (Left, *Rh1>Cdk8 RNAi; UAS-mCherry*) when compared to the control (Middle, *Rh1>Cdk8 RNAi; UAS-CDK19*^*WT*^*)*. Scale bars: 2 μm. **c** Quantification of mitochondrial size and number in photoreceptors (*n* > 50 per genotype for the quantification of mitochondria size, *n* = 7 per genotype for the quantification of mitochondrial number). Quantification of mitochondrial number per photoreceptor terminal (Right) (*n* = 10 per genotype). Statistical analyses are one-way ANOVA followed by a Tukey post hoc test. Results are mean ± SEM (∗*p* < 0.05, ∗∗*p* < 0.01, ∗∗∗∗*p* < 0.0001, ns no significance). Source data are provided as a Source Data file.

To determine mitochondrial size and distribution in photoreceptors (PR) and their synapses, we performed TEM in the retina and lamina upon reduction of *Cdk8* levels using RNAi driven by Rhodopsin1-Gal4 (*Rh1> Cdk8 RNAi*). We found that the size of mitochondria is significantly reduced, and the number of mitochondria is significantly increased in the PR cell body, compared to control (*Rh1* > *UAS-Luciferase RNAi*) and *Cdk8* knockout flies rescued by expression of wild-type human CDK19 (*Rh1>cdk8 RNAi; UAS-CDK19*^*WT*^) (Fig. 3a, c). In addition, the number of mitochondria is reduced in photoreceptor terminals in *Rh1>Cdk8 RNAi* flies when compared to control flies or flies expressing wild-type human CDK19 in the *Cdk8* RNAi background (Fig. 3b). Loss of Drp1 in photoreceptors leads to an increase in small mitochondria in the cell body of PR that are highly clustered as well as a loss of mitochondria at synapses^39^. The data suggested that loss of Drp1 leads mitochondria to be clustered like beads on a string and hence interconnected, and that they form a network that is poorly transported to synapses. These data clearly indicate that *Cdk8* loss partially phenocopies the Drp1 loss phenotypes in photoreceptors. Hence, the loss of Cdk8 impairs mitochondrial fission in PR. Furthermore, these defects in mitochondria can be rescued by expression of wild-type human CDK19.

Given that there is a depletion of mitochondria at synapses as well as a reduction in ATP production, we surmised that there may be a defect in synaptic transmission. Indeed, ref. ^39^ argued that the recycling of synaptic vesicles at synapses requires the presence of functional mitochondria and proper ATP levels to drive the recycling process^40^. We therefore performed Electroretinograms (ERGs) in flies in which *Cdk8* levels are reduced (*elav>Cdk8 RNAi)* and observed a dramatic reduction of on-and-off transients, indicating that the PR fails to communicate with the postsynaptic cells and that synaptic transmission is nearly abolished (Fig. S5a). In contrast, the ERG amplitudes are not altered, suggesting that the phototransduction cascade is not affected (Fig. S5b). These data are strikingly similar to the loss of *Drp1* in photoreceptors^39^.

### Cdk8 and CDK19 have context-dependent subcellular localization

**Fig. 4:**
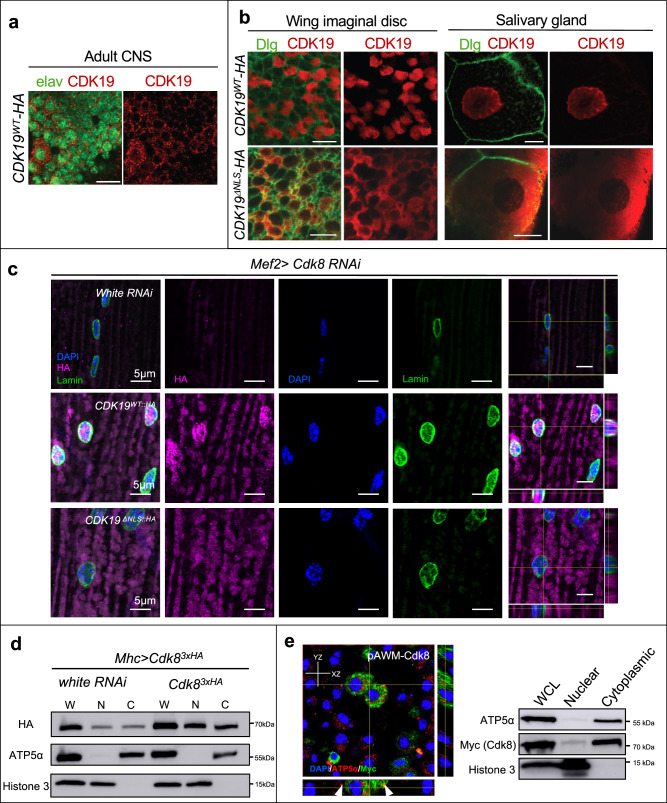
Cdk8 and CDK19 have context-dependent subcellular localizations. **a** Subcellular localization of *UAS-CDK19*^*WT-3xHA*^ in adult CNS. **b** Subcellular localization of *UAS-CDK19*^*WT-3xHA*^ and *UAS-CDK19*^*ΔNLS-3xHA*^ in wing imaginal disks and salivary glands. Green indicates Dlg staining, which marks the cell membrane, and red indicates HA-tagged proteins. The stainings were independently repeated three times. Scale bar: 10 μm. **c** Subcellular localization of *UAS-CDK19*^*WT-3xHA*^ and *UAS-CDK19*^*ΔNLS-3xHA*^ in adult indirect flight muscles. Anti-HA is used to visualize CDK19 localization. DAPI was used to detect nuclear DNA and anti-lamin antibody highlights the nuclear membrane. Cross sections of confocal Z-stacks are shown in the right most column. **d** Myosin heavy chain (Mhc)-Gal4 was used to express Cdk8^3xHA^ in muscles with either control *white* RNAi or an additional copy of Cdk8^3xHA^. Whole cell lysate (W), nuclear (N), and cytoplasmic (C) fractions were prepared, and antibodies to ATP5a and Histone 3 were used to detect the cytoplasmic and nuclear fractions, respectively. **e** Myc-tagged Cdk8 (magenta) localization in S2R+ cells. Co-staining with DAPI and ATP5a highlights cytoplasmic localization. Whole cell lysate (WCL), nuclear, and cytoplasmic fractions were blotted to detect Cdk8. Experiments were repeated three times. Source data are provided as a Source Data file.

We have previously shown that human CDK19^WT^ was primarily localized to cytoplasm in neurons^9^. We thus sought to characterize the localization of CDK19 in several Drosophila tissues. Using an antibody to detect human CDK19, we confirmed the cytoplasmic localization in the adult central nervous system (Fig. 4a), while we observed nuclear localization in larval wing imaginal disks and salivary glands (Fig. 4a). In adult indirect flight muscles, CDK19^WT^ was found in both cytoplasm and nuclei (Fig. 4c).

These findings suggested that CDK19 may play important cytoplasmic roles. To assess the importance of the predicted nuclear localization sequence (NLS) (Fig. S6a, b), we generated *UAS-CDK19 NLS* transgenic flies with and without a 3XHA tag, respectively (*UAS-CDK19*^*ΔNLS-3XHA*^ *and UAS-CDK19*^*ΔNLS*^). Phenotypes produced by both constructs were the same, showing that the 3XHA tag did not affect function. Expression of the tagged construct in wing disks and salivary glands using *da-Gal4* shows that deletion of the NLS leads to a cytoplasmic localization (Fig. 4b). Expression of CDK19^ΔNLS^ in indirect flight muscles showed only cytoplasmic localization. Co-staining with DAPI to detect nuclear DNA and anti-lamin antibodies, which detect the lamins in the nuclear membrane, highlights the exclusion from the nucleus (Fig. 4c). These findings validate that the predicted NLS sequences can target CDK19 to the nucleus.

Given the lack of available antibodies to detect endogenous Cdk8 in tissue, we relied on the HA tags encoded in the Cdk8 transgene to track the localization in subcellular fractions. *Mhc>Cdk8*^3xHA^ flies were crossed to either *white* RNAi or UAS-Cdk8^3xHA^ to produce flies expressing one or two copies of exogenous Cdk8. Nuclear and cytoplasmic fractions were isolated from whole cell lysates, and the presence of Cdk8 was detected with anti-HA antibodies (Fig. 4d). Antibodies to ATP5a were used to highlight the cytoplasmic fraction, while Histone 3 marked nuclear fractions. Cdk8 was found in both nuclear and cytoplasmic fractions, and the relative distribution was unchanged when Cdk8 was elevated. Interestingly, a recent study showed that CDK8 is primarily localized in cytoplasmic in murine pancreatic cells where it regulates insulin secretion^41^, which is in line with our observation on Cdk8.

Finally, to validate our observation, we further expressed Myc-tagged Cdk8 in Drosophila S2R+ cells, which are derived from late-stage embryos^42^, and observed a clear cytoplasmic localization (Fig. 4e, left panel). This distribution was confirmed by western blotting of nuclear and cytoplasmic fractions (Fig. 4e, right panel).

### Endogenous GFP-tagged Cdk8 is found in nuclei and the cytoplasm

**Fig. 5:**
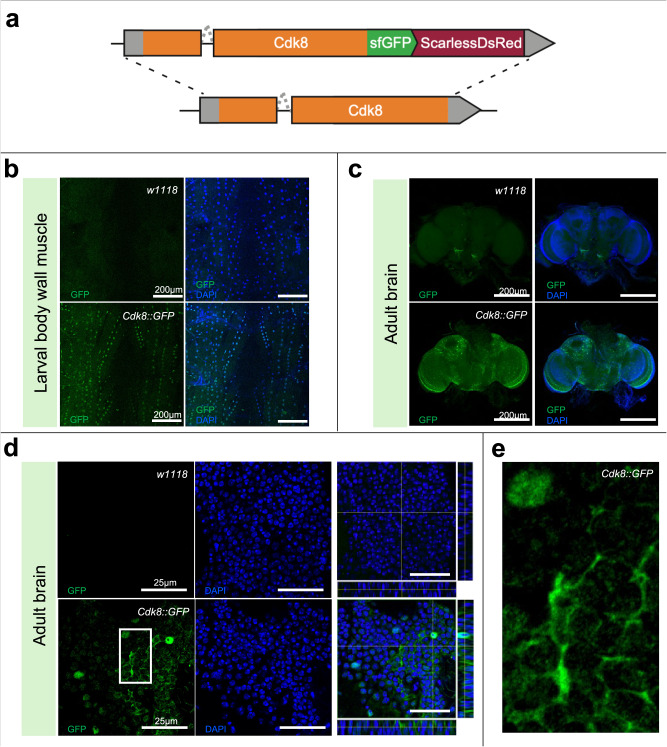
Endogenous GFP-tagged Cdk8 has dynamic subcellular localizations. **a** Schematic illustration for the generation of endogenously tagged *Cdk8::GFP* flies. **b** Subcellular localization of *Cdk8::GFP* in larval body wall muscles in comparison to *w*^*1118*^, as well as in the adult brain. **c** Positive signals rely on the endogenously expressed GFP. *n* = 10 per genotype. **d** Single plane, zoom-in images with orthogonal views of adult brains comparing *w*^*1118*^ and *Cdk8::GFP*. *n* = 10 per genotype. The selected region is shown in (**e**) to further demonstrate the cytoplasmic localization of endogenous Cdk8.

To gain more insight into endogenous Cdk8 localization, we used a CRISPR-based approach to generate a Cdk8-superfolder-GFP fusion protein fly line (referred to as Cdk8::GFP) in which the resulting endogenous Cdk8 protein is tagged with superfolder GFP (Fig. 5a)^43^. The construct also encodes a scarless-DsRed in the untranslated region of *Cdk8*, which aids in the selection of transformed flies. *Cdk8::GFP* homozygous flies are viable and do not exhibit any overt phenotypes, indicating that insertion of sfGFP does not affect Cdk8 function. Through this GFP insertion, the detection of GFP reflects the endogenous Cdk8 protein expression and localization. In these experiments, we detected the GFP fluorescence without the use of anti-GFP antibodies, to eliminate any chance of non-specific staining. In larval body wall muscles, Cdk8::GFP is primarily nuclear, though low-level cytoplasmic staining is seen throughout the tissue when compared to the control (Fig. 5b). In adult brains, Cdk8 shows a dynamic distribution (Fig. 5c) characterized by nuclear and cytoplasmic staining (Fig. 5d). Some cells show primarily nuclear while others show primarily cytoplasmic localization (Fig. 5e), highlighting the variability in Cdk8 protein localization and demonstrating in vivo that endogenous Cdk8 is present in the cytoplasm. We used fractionation of adult brain protein lysates to further validate the cytoplasmic localization of Cdk8::GFP (Fig. S7a).

### Cdk8 interacts with Drp1 and promotes its phosphorylation and mitochondrial fragmentation

Given that the variants observed in human CDK19 patients are enriched in the kinase domain^9^ and since Cdk8^KD^ acts as the dominant negative variant when expressed in flies, we assume that the kinase function of Cdk8 may regulate mitochondrial dynamics. We performed protein sequence alignments for the CDK consensus phosphorylation motif S/T-P-X-R/K^44^ for regulators of mitochondrial dynamics, including Opa1, Marf, and Drp1^45^. We did not find the motif in the fusion proteins (Opa1and Marf), but the fission regulator Drp1 carries the motif at serine 616 (Fig. S5c), a site that is known to be regulated by phosphorylation, acetylation, and SUMOylation^46^. These findings suggest that the effects of mitochondrial dynamics of Cdk8 may be mediated by the fission regulator, Drp1.

**Fig. 6:**
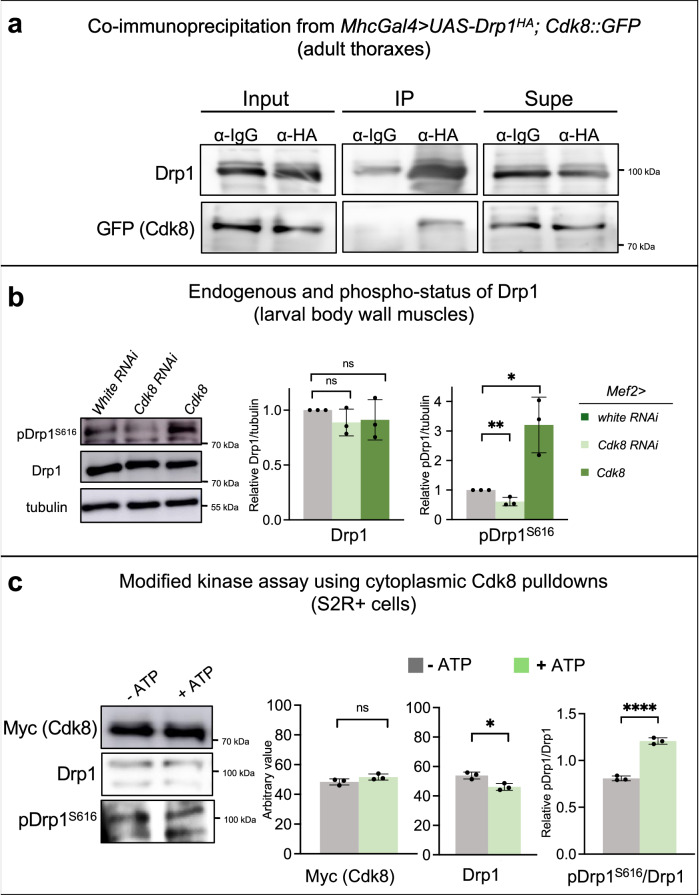
Cdk8 interacts with Drp1 and phosphorylates Drp1 at S616. **a** Co-immunoprecipitation using adult thoraxes expressing *MhcGal4* > *UAS-Drp1*^*HA*^*+Cdk8::GFP* at 25 °C. IgG agarose beads were used as a negative control. IPs used HA-agarose beads and detected endogenous Cdk8::GFP using rabbit anti-GFP antibody. Experiments were individually repeated three times. **b** Western blot showing the level of total Drp1 or S616 phospho-Drp1 in control (*Mef2>white RNAi*), *Mef2>Cdk8-RNAi* and *Mef2>Cdk8*^*HA*^flies. Quantifications of blots normalized to actin levels (*n* = 3 per genotype). Results are mean ± SD. An unpaired two-tailed *t*-test is used to generate the statistics. *P* value is 0.1850 for *Cdk8 RNAi*, and 0.4490 for *Cdk8* in comparison to *white RNAi*. Flies were raised at 25 °C. **c** Myc-Cdk8 and HA-Drp1 from S2R+cytoplasmic fractions were immunoprecipitated with Myc-agarose and subjected to in vitro kinase assays in the presence or absence of ATP. Experiments are repeated three times. Results are mean ± SD. An unpaired two-tailed *t*-test is used to generate the statistics. *P* value is 0.0149 for Drp1 and 8.54e-05 for pDrp1/Drp1. Samples were probed with antibodies to Myc to quantify Myc-Cdk8 levels (which were equivalent between conditions), Drp1 to detect the degree of Drp1 binding to Cdk8, and with anti-pDrp1S616 to quantify the degree of phosphorylation relative to total Drp1. ∗*p* < 0.05, ∗∗*p* < 0.01, ∗∗∗∗*p* < 0.0001, ns no significance. Source data are provided as a Source Data file.

To test if endogenous GFP-tagged Cdk8 can physically interact with Drp1, we performed co-immunoprecipitation using fly muscles expressing HA-tagged Drp1 in the Cdk8::GFP fly strain (Fig. 6a). Drp1 was pulled down using anti-HA beads and Cdk8::GFP was detected using an anti-GFP antibody in co-immunoprecipitations of Drp1 but not with anti-IgG agarose. This finding shows that Drp1, which is cytoplasmic, can interact with endogenous GFP-tagged Cdk8. We also performed co-immunoprecipitation using S2R^+^ insect cells co-transfected with Myc-tagged Cdk8 and HA-tagged Drp1 plasmids (Fig. S7b). Drp1 was pulled down with Cdk8 in the Myc-tagged Cdk8 immunoprecipitation (IP), while Drp1 was absent from the control IgG IP. Cdk8 was also pulled down in the reciprocal pulldown using HA-tagged Drp1 (Fig. S7b). These findings show that Cdk8 physically interacts with Drp1.

We next asked if Cdk8 expression affects the abundance of Drp1 or S616-phosphorylated Drp1. We examined the levels of both endogenous total Drp1 and phospho-S616-Drp1 in lysates from fly tissues in which *Cdk8* was either knocked down or overexpressed in larval body wall muscles. We found no significant difference in the level of Drp1 protein between the control and either Cdk8-RNAi or Cdk8 overexpression (Fig. 6b). However, an antibody that recognizes pDrp1^S616^, shows a significant decrease following *Cdk8* depletion and an increase in pDrp1^S616^ when Cdk8 is expressed (Fig. 6b). To determine if loss of *Cdk8* affects the phosphorylation of Drp1 in neurons, we assessed the protein levels of phospho-Drp1 S616 and normal Drp1 in adult heads by performing Western blot using the extracts of heads of *elav>Cdk8 RNAi* flies. We found that the phospho-Drp1 S616 level is decreased by about 25% (Fig. S5d). In summary, the data show that Cdk8 interacts with Drp1 and promotes phosphorylation of Drp1 at S616, and activates the mitochondrial fragmentation process. Our data strongly suggest that the effect of Cdk8 on Drp1 is direct, although we cannot rule out entirely that it is indirect via an associated kinase.

Given that Cdk8 and Drp1 interact and are both cytoplasmic, we immunoprecipitated Myc-Cdk8 from the cytoplasmic fractions of S2R+ cells transfected with both Myc-Cdk8 and HA-Drp1 constructs. The immunoprecipitated proteins were then subjected to in vitro kinase assays in the absence or presence of ATP (Fig. 6c). We quantified the total amount of Cdk8, which was equivalent between experimental conditions. We next determined relative amounts of total Drp1 and pS616-Drp1. We found that phosphorylation of co-immunoprecipitated cytoplasmic Drp1 is significantly elevated in the presence of ATP (Fig. 6c).

### Cytoplasmic CDK19 replaces the function of Cdk8 in neurons and rescues mitochondrial defects

**Fig. 7:**
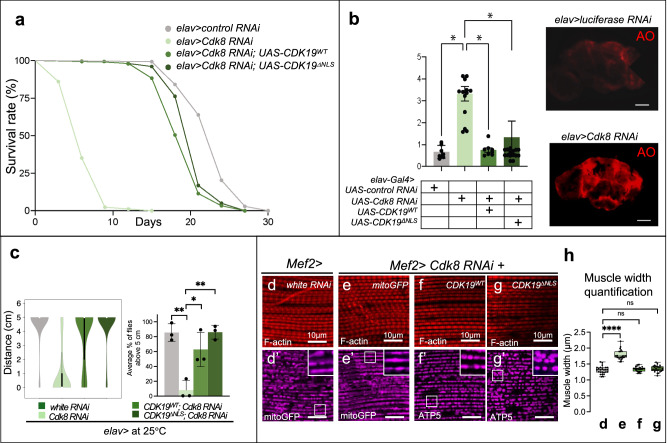
Expression of reference CDK19^WT^ or CDK19^∆NLS^ can rescue both muscle and mitochondrial defects caused by Cdk8 depletion. **a** *CDK19* ^*ΔNLS*^ can suppress the lifespan decrease caused by an expression of *Cdk8 RNAi* in the neuron to a similar extent to *CDK19*^*WT*^ expression (*n* > 100 per genotype). **b** Quantification of acridine orange staining (AO), which assesses neuronal death. when Cdk8 is decreased in adult brain neurons. Red indicates the dying neurons. (*n* = 7, *n* = 14, *n* = 8, and *n* = 16 respectively in order). Scale bar: 10 μm. Statistical analyses are one-way ANOVA followed by a Tukey post hoc test. Results are mean ± SEM (∗*p* < 0.05, ∗∗*p* < 0.01, ∗∗∗∗*p* < 0.0001, ns no significance). **c** Both *CDK19*^*WT*^ and *CDK19* ^*ΔNLS*^ can significantly suppress the climbing defects due to neuronal depletion of cdk8 (*elav>Cdk8 RNAi*). Experiments are repeated four times. Results are mean ± SD. An unpaired two-tailed *t*-test is used to generate the statistics. *P* value is 4.56e-05 for white RNAi, 2,38e-05 for CDK19^WT^, and 0.0036 for CDK19^ΔNLS^ in comparison to *Cdk8 RNAi*. The total number of progeny are assessed, *n* = 160, 153, 140, 159, respectively, in order. **d**–**g** Rhodamine-phalloidin staining (F-actin, red) to visualize indirect flight muscles in (**d**) control *white* RNAi or (**e**) *Cdk8* RNAi flies. **f** CDK19^WT^ and (**g**) CDK19^ΔNLS^ were expressed in the *Cdk8*-depleted background. **d**’–**g**’ Either mitoGFP or ATP5α staining (magenta) were used to visualize mitochondrial morphology in the indicated genotypes. Scale bars: 10 μm. **h** Quantification of adult indirect flight muscle width in 3-day-old flies. *n* = 5 per genotype. Data were presented as box plots (center line at the median, upper bound at 75th percentile, lower bound at 25th percentile) with whiskers at minimum and maximum values. An unpaired two-tailed *t*-test is used to generate the statistics. *P* value is 1.78e-16 for *white RNAi*, 0.2295 for *CDK19*^*WT*^, and 0.1816 for *CDK19*^*ΔNLS*^ in comparison to *Cdk8 RNAi*. All raised at 25 °C. ∗*p* < 0.05, ∗∗*p* < 0.01, ∗∗∗∗*p* < 0.0001, ns no significance. Source data are provided as a Source Data file.

The data presented so far in muscles and neurons argue that Cdk8 and CDK19 function in the cytoplasm to promote mitochondrial fission by phosphorylating Drp1. However, in muscles as well as in the developing third instar larval brains Cdk8 is present in nuclei and the cytoplasm, begging the question of whether the cytoplasmic localization is sufficient for this function^9^. To determine if an expression of cytoplasmic CDK19 can rescue the loss of Cdk8 in the nervous system, we expressed *Cdk8 RNAi* in neurons (*elav>Cdk8 RNAi*) and examined the effects of expressing either the reference *CDK19* (*UAS-CDK19*^*WT*^) or *UAS-CDK19*^*ΔNLS*^. As shown in Fig.7a, expression of *CDK19*^*ΔNLS*^ is sufficient to rescue the lifespan defects observed in flies expressing *Cdk8 RNAi* in neurons (*elav>Cdk8 RNAi)* similar to CDK19^WT^ expression (Fig. 7a).

We next assessed cell death induced by *elav>Cdk8 RNAi* in the brain in 10-day old flies kept at 25 °C by staining with acridine orange (AO) and quantified fluorescence intensity. We observed that an elevated level of AO can be significantly reduced by expression of either *UAS-CDK19*^*WT*^ or *UAS-CDK19*^*ΔNLS*^ (Fig. 7b). Depletion of Cdk8 in neurons leads to impaired climbing ability (Fig. 1b) which can also be rescued by expression of either *UAS-CDK19*^*WT*^ or *UAS-CDK19*^*∆NLS*^ (Fig. 7c). These findings argue strongly that the function of Cdk8 in these assays is cytoplasmic.

We also examined the effects of wildtype and cytoplasmic CDK19 in muscles. To visualize mitochondrial morphology, we used either mitoGFP or an antibody against ATP5α (Fig. 7d’–g’), whereas muscle fibers were visualized using rhodamine-phalloidin to detect filamentous actin (Fig. 7d–g). *Cdk8* knockdown leads to a widening of muscle fibers compared to control (Fig. 7d–h). Expression of either UAS-CDK19^WT^ or UAS-CDK19^∆NLS^ restored muscle fiber morphology equally well (Fig. 7f–h). We next examined mitochondrial morphology. We observed that expression of both *UAS-CDK19*^*WT*^ or *UAS-CDK19*^*∆NLS*^ in a wild-type background led to rounded and fragmented mitochondria, indicating they can promote fission (Fig. S3l’, m’). A mixture of elongated and fragmented mitochondria was found in control (Fig. 7d), and depletion of *Cdk8* caused a highly elongated mitochondrial morphology (Fig. 7e). We observed predominantly fragmented mitochondria when either UAS-CDK19^WT^ or UAS-CDK19^∆NLS^ was co-expressed in *Mef2>Cdk8 RNAi* background (Fig. 7f’, g’). These results show that cytoplasmic CDK19 is sufficient to regulate mitochondrial morphology.

### Expression of Cdk8 can rescue *pink1* muscle degeneration and locomotor defects

**Fig. 8:**
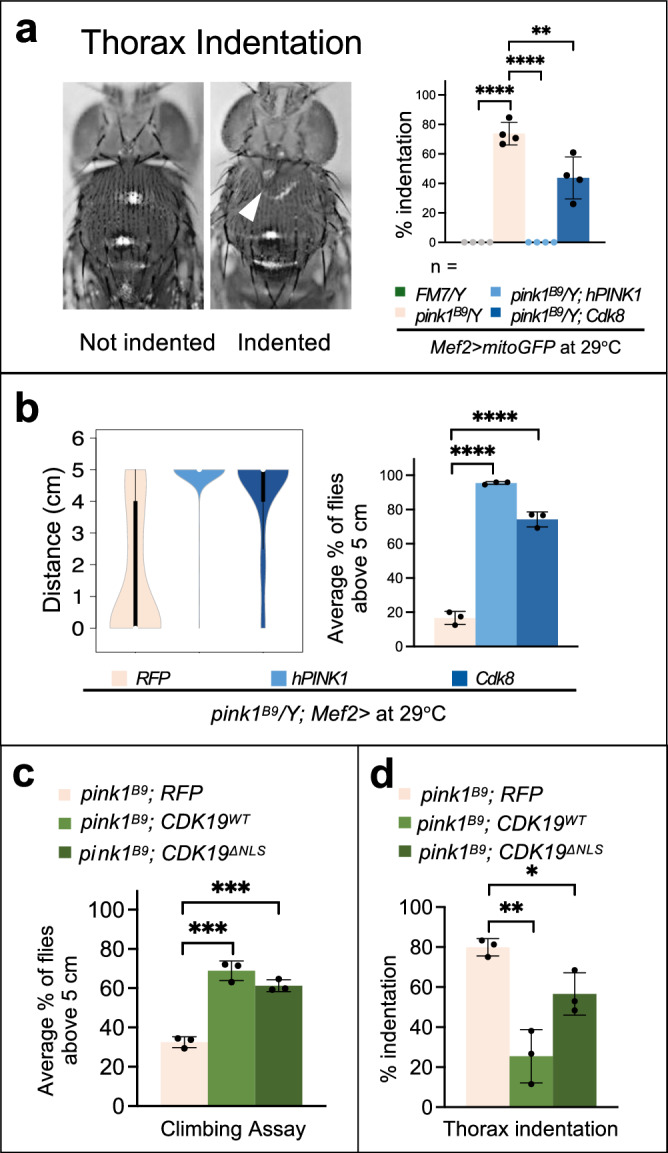
Elevated Cdk8 and CDK19 can rescue the defects in thorax integrity and climbing ability in *pink1*^*B9*^mutant flies. **a** Thorax indentation (white arrowhead indicates indentation) with quantification of percent indentation in 3-day-old flies expressing either *RFP, hPink1*, or *Cdk8* with *Mef2-Gal4* in the *pink1*^*B9*^ mutant background compared to FM7/Y control flies. Experiments are repeated four times. The total number of progeny are assessed, *n* = 120, 130, 137, 119, respectively, in order. Results are mean ± SD. An unpaired two-tailed *t*-test is used to generate the statistics. *P* value is 1.30e-6 for both *FM7/Y* and *hPINK1* and 0.01 for *Cdk8* in comparison to the *pink1*^*B9*^ mutant. **b** Violin plots showing the distribution of climbing activity of *RFP, hPink1,* or *Cdk8* with *Mef2-Gal4* in the *pink1*^*B9*^ mutants. The average percent of flies that reach the target line in indicated genotypes. Experiments are repeated three times. The total number of progeny are assessed, *n* = 80, 74, 65, respectively, in order. Results are mean ± SD. An unpaired two-tailed *t*-test is used to generate the statistics. *P* value is 3.96e-6 for *hPINK1* and 6.62e-5 for *Cdk8* in comparison to the *pink1*^*B9*^ mutant. Raised at 29 °C. **c** Climbing assay testing the ability of RFP, CDK19^WT^, or CDK19^∆NLS^ expressed in the *pink1*^*B9*^ background to reach the target line. Experiments are repeated three times. The total number of progeny are assessed, *n* = 56, 71, 98, respectively, in order. Results are mean ± SD. An unpaired two-tailed *t*-test is used to generate the statistics. *P* value is 0.0004 for *CDK19*^*WT*^ and 0.0002 for *CDK19* ^*ΔNLS*^ in comparison to the *pink1*^*B9*^ mutant. **d** Quantification of thorax indentation in flies when RFP, CDK19^WT^, or CDK19^ΔNLS^ are expressed in the *pink1*^*B9*^ background, raised at 25 °C. Experiments are repeated three times. Total number of progeny are assessed, *n* = 56, 77, 84. Results are mean ± SD. An unpaired two-tailed *t*-test is used to generate the statistics. *P* value is 0.0025 for CDK19^WT^ and 0.0238 for CDK19 ^ΔNLS^ in comparison to the *pink1*^*B9*^ mutant. ∗*p* < 0.05, ∗∗*p* < 0.01, ∗∗∗*p* < 0.001, ∗∗∗∗*p* < 0.0001. Source data are provided as a Source Data file.

Our results so far show that Cdk8 regulates mitochondrial fission by phosphorylating Drp1. Interestingly, Pink1 was previously shown to directly phosphorylate Drp1 at S616 to promote its activity and fragmentation of mitochondria^47,48^. Given that both Cdk8 and Pink1 phosphorylate the same amino acids of Drp1 and given that they are both required for normal mitochondrial function neither alone may be sufficient to fully phosphorylate Drp1 at S616. Hence, overexpression of *Cdk8* may be able to suppress the phenotypes associated with the *pink1* nulls (*pink1*^*B9*^), a fly model for PD^17^. *pink1* is on the X chromosome and hemizygous *pink1*^*B9*^ males were used in subsequent experiments. Loss of *pink1* causes thorax indentation (Fig. 8a), an established indicator for the underlying muscle degeneration^17^. Muscle degeneration is prominent in *pink1* or *parkin* mutant flies where apoptotic cell death leads to thorax indentation and flight and mobility defects^17,21^. Approximately 90% of *pink1*^*B9*^ flies have thorax indentation, and expression of human Pink1 (hPink1) using the muscle driver, *Mef2-Gal4*, fully rescues the phenotype (Fig. 8a). *Mef2>Cdk8* expression also significantly rescued the phenotype as only 40% of flies showed thorax indentation defects (Fig. 8a).

Next, we tested the effect of Cdk8 expression on the severe locomotor defects observed in *pink1*^*B9*^ flies (Fig. 8b). *pink1*^*B9*^ climbing defects were significantly rescued by expression of either hPINK1 or Cdk8 in 3-day old flies (Fig. 8b). Thorax indentation (Fig. S8a) as well as climbing ability (Fig. S8b) were also examined in 3-week-old flies. Both hPINK1 and Cdk8 significantly rescued these phenotypes in these aged flies. In summary, Cdk8 expression can significantly rescue thorax indentation and climbing defects in *pink1*^*B9*^ mutants.

### Cytoplasmic CDK19 can rescue *pink1*^*B9*^ phenotypes

We observed that either overexpression of CDK19^WT^ or CDK19^∆NLS^ is sufficient to induce severe mitochondrial fragmentation, as observed with the expression of Cdk8. These results led us to wonder if their expression could rescue the *pink1*^*B9*^ mutant phenotypes, similar to the expression of Cdk8. We assayed the climbing activity (Fig. 8c) and thorax indentation (Fig. 8d) phenotypes when either CDK19^WT^ or CDK19^∆NLS^ were expressed in a *pink1*^*B9*^ mutant background. Strikingly, both wildtype and cytoplasmic CDK19^∆NLS^ significantly rescued both phenotypes (Fig. 8c, d). These findings show that the functions of Cdk8 are conserved in cytoplasmic CDK19.

### Cdk8 expression rescues mitochondrial defects and muscle degeneration in *pink1*^*B9*^ flies

**Fig. 9:**
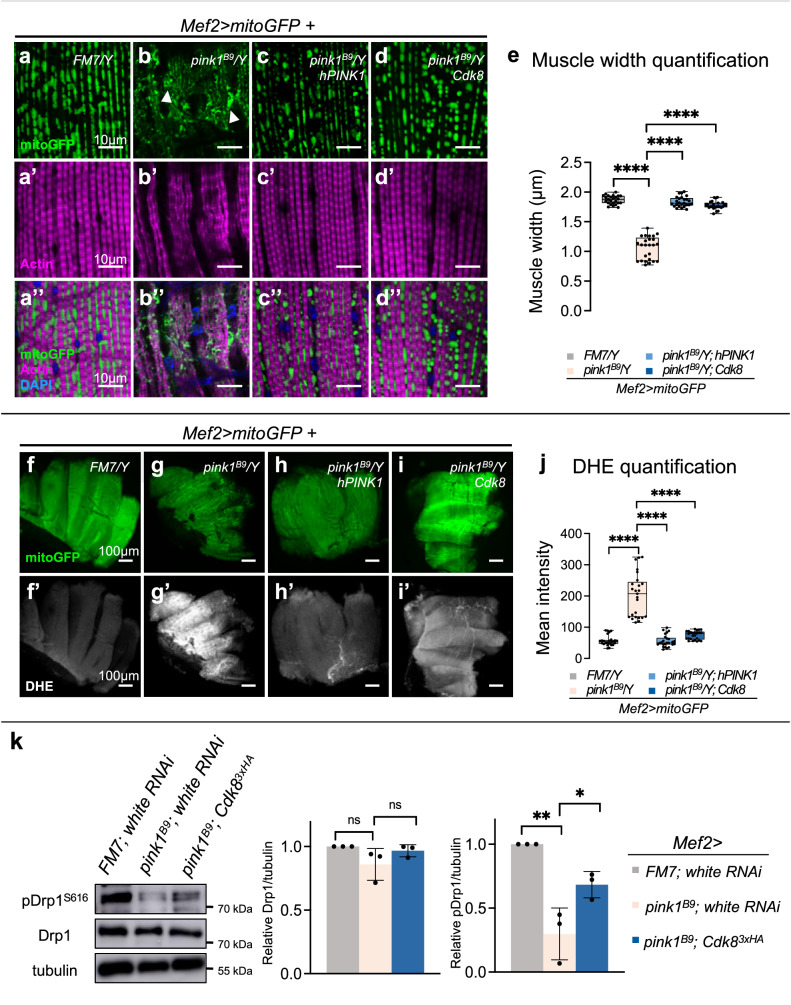
Cdk8 can rescue *pink1*^*B9*^ mitochondrial and muscle defects, and expression of cytoplasmic CDK19 rescues *pink1*^*B9*^ behavior defects. **a**–**d** Mitochondrial morphology in indirect flight muscle (IFM) in (**a**) *FM7/Y*, (**b**) *pink1*^*B9*^*/Y*, and either (**c**) hPINK1, or (**d**) Cdk8 expressed in the *pink1*^*B9*^ mutant background with *Mef2>mitoGFP*. **a**′–**d**′ Rhodamine-phalloidin staining (magenta) stains actin filaments to visualize the muscle morphology. **a**″–**d**″ Merged images, including DAPI staining for DNA (blue). Scale bars: 10 μm. **e** Quantification of adult indirect flight muscle width in 3-day-old flies (*n* = 5 per genotype). Data were presented as box plots (center line at the median, upper bound at 75th percentile, lower bound at 25th percentile) with whiskers at minimum and maximum values. An unpaired two-tailed *t*-test is used to generate the statistics. *P* value is 1.13e-024 for *FM7/Y*, 1.75e-23 for *hPINK1*, and 1.42e-22 for *Cdk8* in comparison to the *pink1*^*B9*^ mutant. **f**–**i** IFM in (**f**) *FM7/Y*, (**g**) *pink1*^*B9*^, and (**h**) hPink1 or (**i**) Cdk8 expressed in the *pink1*^*B9*^ mutant background with *Mef2>mitoGFP* raised at 29 °C. **f**′–**i**′ DHE staining of corresponding genotypes in (**f**–**i**). Scale bars: 100 μm. **j** Quantification of mean DHE fluorescence intensity in live adult IFM of the indicated genotypes (*n* = 8 per genotype). Data were presented as box plots (center line at the median, upper bound at 75th percentile, lower bound at 25th percentile) with whiskers at minimum and maximum values. An unpaired two-tailed *t*-test is used to generate the statistics. *P* value is 3.11e-25 for *FM7/Y*, 1.71e-26 for *hPINK1*, and 1.46e-12 for *Cdk8* in comparison to the *pink1*^*B9*^ mutant. All raised at 29 °C. **k** Western blot showing the level of total Drp1 or S616 phospho-Drp1 in control (*Mef2>white RNAi*), *pink1* mutant flies (*pink1*^*B9*^; *Mef2>white RNAi)* and *pink1* expressing Cdk8 (*pink1*^*B9*^; *Mef2>Cdk8*^3xHA^. Quantifications of blots normalized to actin levels. (*n* = 3 per genotype). Results are mean ± SD. An unpaired two-tailed *t*-test is used to generate the statistics. *P* value is 0.0039 for FM7 and 0.0424 for Cdk8 in comparison to *pink1*^*B9*^ mutant for detecting the level of pDrp1^S616^. ∗*p* < 0.05, ∗∗*p* < 0.01, ∗∗∗∗*p* < 0.0001, ns no significance. Source data are provided as a Source Data file.

We next examined the muscle and mitochondrial morphology in dorsal longitudinal adult indirect flight muscles in the thorax^49,50^. We used mitoGFP to detect mitochondria and rhodamine-phalloidin to visualize F-actin and overall muscle integrity in dissected IFMs. In control male flies (FM7/Y), elongated and tubular mitochondria (Fig. 9a) are found evenly distributed and intercalated between well-defined muscle fibers (Fig. 9a”). Mitochondrial morphology was severely disrupted in *pink1*^*B9*^ mutants as previously reported (Fig. 9b)^16,17,21^: they exhibit a reduced number of mitochondria, irregularly shaped mitochondria that are scattered between the muscle fibers, and the muscle fibers are significantly narrower in width than in control animals (Fig. 9b’)^16,17,20,21,23,51^. Aggregates of mitochondria are also seen (white arrowheads in Fig. 9b)^52,53^. Expression of both hPINK1 and Cdk8 rescued the defects in both mitochondria and muscle (Fig. 9c, d”). Myofibril width quantification revealed that expression of either hPINK1 or Cdk8 in *pink1*^*B9*^ mutants significantly suppressed muscle degeneration (Fig. 9e). Together, these findings show that elevated expression of Cdk8 can suppress mitochondrial and muscle defects observed in the *pink1*^*B9*^ PD model.

### Cdk8 suppresses ROS production in *pink1*^*B9*^ mutants

Loss of Pink1 causes accumulation of dysfunctional mitochondria, which impairs the electron transport chain (ETC) and, in turn, leads to elevated production of reactive oxygen species (ROS)^54^. Since an expression of Cdk8 can alleviate the mitochondrial phenotypes in *pink1*^*B9*^, we asked if the morphologically restored mitochondria are also functionally restored. We used dihydroethidium (DHE) staining to assess ROS in adult indirect flight muscles^55^. The FM7/Y control males show redox homeostasis under physiological conditions (Fig. 9f, f’). In contrast, *pink1*^*B9*^ flies show enhanced DHE intensity, indicative of elevated ROS production^56,57^ (Fig. 9g, g’), and elevated DHE staining is observed near aggregated mitochondria (Fig. S8d, d”, yellow arrowhead). hPINK1 significantly reduced mitochondrial aggregation and suppressed ROS elevation (Fig. 9h and S8e, e’). ROS production in Cdk8-expressing *pink1*^*B9*^ flies was also reduced, and mitochondrial integrity was restored (Fig. 9h’ and S8f-f”). Quantification of DHE mean intensity demonstrated that either hPINK1 or Cdk8 significantly suppressed the elevated ROS levels caused by *pink1*^*B9*^ mutants (Fig. 9j). We also used a spectrophotometer to measure emitted DHE fluorescence from adult thoraxes (Fig. S8g), confirming that elevated ROS found in *pink1*^*B9*^ flies is significantly suppressed by expression of either hPINK1 or Cdk8.

### Cdk8 rescues *pink1*-induced decreases in phospho-Drp1

Our results suggest that Cdk8 can promote Drp1 activity through phosphorylation and mitochondrial fission. Since Pink1 can also carry out this role, we asked whether, in the absence of functional Pink1, elevated expression of Cdk8 could restore Drp1 phosphorylation levels. Using fly lysates from control flies, *pink1* mutants, and *pink1* expressing wild-type Cdk8, we found that the overall levels of endogenous Drp1 were similar in all three samples (Fig. 9k). In contrast, *pink1* mutants showed significant decreases in pDrp1S616, relative to control, and these decreased levels were significantly rescued by expression of Cdk8 (Fig. 9k).

## Discussion

Here we show that depletion of *Cdk8* in muscle causes a severe reduction of mitochondrial fission, a phenotype that is also observed when Drp1 is lost. We show that Cdk8 binds to Drp1, phosphorylates Drp1 at S616, and that loss of *Cdk8* leads to decreased phosphorylation of Drp1 S616 in muscles and neurons. Interestingly, *pink1* also phosphorylates Drp1 at S616, and loss of *pink1* causes defects in mitochondrial dynamics, mitophagy, and Complex I function. Overexpression of *Cdk8* rescues phenotypes associated with loss of *pink1*, including locomotor impairment and defects in mitochondrial morphology and muscle integrity. The data indicate that Cdk8 functions similarly to Pink1. We demonstrate that endogenous Cdk8 can localize to the cytoplasm in muscles and brain supporting our model that Cdk8 has cytoplasmic functions. In summary, our data reveal a new cytosolic function for Cdk8/CDK19 in mitochondrial fission by mediating the phosphorylation of Drp1 and this function parallels the function of *pink1*.

### Functional conservation of Cdk8 and CDK19

CDK8 and its paralog, CDK19, are cyclin-dependent kinases known to regulate transcription. Either CDK8 or CDK19 are components of a 4-protein complex called the Mediator kinase module^1^. However, they are mutually exclusive in this complex. This module reversibly associates with Mediator, a 26-subunit protein complex that regulates RNA Polymerase II-mediated gene expression. As part of this complex, the other Mediator kinases have been implicated in diverse processes such as developmental signaling, metabolic homeostasis, and innate immunity^58^. In recent years, dysregulation of Mediator kinase module proteins, including CDK8/19, have been implicated in the development of different human diseases, in particular, cancer^59^.

In mice and humans, CDK8 is expressed ubiquitously, whereas CDK19 is enriched in the brain^60^. Loss of *CDK8* results in embryonic lethality in mice^61^, whereas loss of *CDK19* causes a mild phenotype (International Mouse Phenotyping Consortium; https://www.mousephenotype.org/). The latter mice are born at expected Mendelian ratios, and the only obvious phenotype is an increase in grip strength. In contrast, in humans, variants in *CDK19* act as dominant negative variants and cause developmental delay, infantile spams, hypotonia, and intellectual disability^9^. Several *CDK8* variants located in the kinase domain also behave as dominant negative alleles and cause hypotonia, facial dysmorphisms, and congenital heart disease^7,8^. Thus far, all phenotypes associated with *CDK8* and *CDK19* are thought to be related to their nuclear function.

We previously observed that human CDK19 is localized to the cytoplasm in adult fly neurons^9^. Moreover, flies that have reduced Cdk8 protein in most cells displayed phenotypes that were strikingly similar to the ones observed in *pink1* null mutants^17,62^. Importantly, overexpression of Cdk8 significantly suppresses the behavioral and mitochondrial defects associated with the loss of *pink1*, which encodes a putative serine/threonine kinase with a mitochondrial targeting sequence. Pink1 promotes mitochondrial clearance, and phosphorylates Drp1 at site S616 in mice and human fibroblast^47^. Hence, these data implicate CDK19/Cdk8 in a critical mitochondrial function.

### Regulation of Drp1 by Cdk8/CDK19 and PINK1

Perturbations in mitochondrial morphology have been associated with several human disorders^63^. Some of the genes are involved in regulating fission and fusion^64^. Mitofusins (Mfn1 and Mfn2) regulate mitochondrial outer membrane fusion and are required to maintain a mitochondrial network in cells, while mitochondrial inner membrane protein Opa1 regulates mitochondrial inner membrane fusion^65^. Drp1, a cytosolic dynamin GTPase, is the key player in mitochondrial fission. Importantly, Drp1 phosphorylation plays a critical role in controlling mitochondrial fission^66^. Phosphorylation of Drp1 at the S616, S579, and S600 sites promotes fission during mitosis and oxidative stress^67–70^. We show that Cdk8 upregulates the phosphorylation of Drp1 at S616 and hence acts in a very similar fashion as Pink1. This observation, combined with the fact that overexpression of Cdk8 nearly fully suppresses climbing and mitochondrial phenotypes associated with loss of *pink1* strongly argues that Cdk8 plays a critical and similar role in mitochondrial dynamics as Pink1. Hence, it is likely that another downstream effector of Pink1, Parkin, may also be affected by Cdk8/CDK19. However, loss of *pink1* also affects iron-sulfur cluster synthesis, which leads to a cascade of events that are independent of mitophagy, and these does not require Parkin^62^.

### Drp1 and human diseases

DNM1L (Dynamin 1-like, synonyms Drp1) is a human homolog of Drp1, which is a member of the dynamin superfamily of GTPases. DNM1L mediates mitochondrial as well as peroxisomal fission^71^, and a patient with loss of *DNM1L* presented with a lethal encephalopathy due to defective mitochondrial and peroxisomal fission (MIM #614388)^72^. This individual had poor feeding, poor growth, lactic acidosis, seizures, hypotonia, nystagmus, and an abnormal gyral pattern on magnetic resonance imaging and passed away at 37 days of life^71^. Mice lacking Dlp1, the homolog of DNM1L, die at embryonic day E12.5, indicating a crucial role for this gene in mammalian development. These mice display abnormal mitochondrial and peroxisomal morphology^73^, indicating that Drp1 is critical in development across the species. The human CDK19 patients show some similar but milder phenotypes, including developmental delay, seizure, and hypotonia, suggesting that the function of DNM1L is not fully dependent on Pink1 and CDK19.

### Mitochondrial dynamics under physiological conditions associated with the translocation of CycC and CDK19

Cyc C is a binding partner of CDK8/CDK19. Under normal conditions, CDK19-Cyc C regulates transcription through association with the Mediator complex of RNA polymerase II^2^. However, in contrast to other cyclins, the concentration of Cyc C does not change during cell-cycle progression^74–76^. Instead, previous work has shown that oxidative stress can induce a nuclear release of Cyc C to the mitochondria, where it facilitates fission through Drp1 and Mff in mouse embryonic fibroblasts^35^. Here we show that expression of Cdk8/CDK19 can modulate mitochondrial morphology under physiological conditions, likely through regulating Drp1, in a similar fashion as Cyc C. We found that the effects of Cdk8 depletion could not be rescued by ectopic Cyc C, suggesting that under our experimental conditions, the two proteins may act on Drp1 through distinct mechanisms.

### Roles of CDK8/19 in rare diseases and implications for common disorders like Parkinson’s Disease

Patients harboring missense mutations in either CDK8 or CDK19 present with a range of clinical features, including neurodevelopmental defects, variable intellectual disability, hypotonia, and facial dysmorphology^7–10^. Thus far, the features of these rare syndromes have been attributed to dysfunction of these kinases as part of the Mediator kinase module. We propose that aspects of these syndromes may also be due to altered function in the cytoplasm in the regulation of mitochondrial morphology and function through effects on Drp1, independent of their role in mediating transcription. In this study, we show that loss of *Cdk8* causes defects that show some similarity to Parkinsonism and both Cdk8 and CDK19 can suppress the phenotypes associated with fly *pink1* mutants. These findings suggest CDK8/19 may also contribute to aspects of the development of Parkinson’s disease.

## Methods

### *Drosophila* culture

*Drosophila melanogaster* flies were raised on standard cornmeal-molasses food. Stocks were kept at 25 °C. Crosses were carried out at 25 and 29 °C as indicated. Since Gal4 activity is elevated at higher temperatures^77^, we could modulate expression levels using both temperatures. Results found at 29 °C were more penetrant, so the main figures include these data, and supplemental figures show data from 25 °C. The following fly strains were used: *Act5c-Gal4/TM6B* (BL #3954), *Mef2-Gal4* (BL #27390), *Appl-Gal4* (BL #32040), *pink1*^*B9*^ (BL #34749), *UAS-Cdk8-RNAi* (BL #35324), *UAS-RFP* (BL #7119), *UAS-white-RNAi (BL #33762)*, *UAS-mito-HA-GFP* (abbreviated as *UAS-mitoGFP*; BL #8442), *UAS-hPINK1-FLAG* (BL #52004), *UAS-Cdk8*^*ORF*^ which expresses a full-length open reading frame (F001348), *UAS-Cdk8-RNAi* (BL #67010), *UAS-Cdk8-3xHA* which expresses a full-length HA-tagged Cdk8 (F001713), *UAS-Cdk8*^*KD*^ was obtained from Dr. J-Y Ji from Tulane University School of Medicine, *UAS-CycC-RNAi* (BL #33753), *UAS-CycC-3xHA* which expresses a full-length HA-tagged CycC (F003051), UAS-luciferase RNAi (BL #35788), UAS-CDK19^WT^, UAS-CDK19^Y32H^ and UAS-CDK19^T196A^ were generated in the previous study (Chung et al. ^9^). Strains with BL stock number were obtained from Bloomington Drosophila Stock Center (Bloomington, IN, USA). Strains labeled with an F initial were obtained from FlyORF Drosophila Stock Center (ZurichORFeome Project, Zurich, Switzerland).

### Documentation of adult *Drosophila* phenotypes

Adult flies were anesthetized with CO_2_ gas and photographed on a Leica MZ6 dissecting stereo microscope with an attached Leica IC90 E digital camera.

### Generation of UAS-CDK19^*ΔNLS*^ transgenic flies

*UAS-CDK19*^*ΔNLS*^ transgenic flies were generated as previously described^78^. Site-directed mutagenesis was performed with the Q5 site-directed mutagenesis kit (NEB), followed by Sanger verification. The following forward and reverse primers were used to make *CDK19*^*ΔNLS*^ or *CDK19*^*ΔNLS −3xHA*^

FW- CGGCTAGGGCCTTCAGGC

RV- GTTTGGAGGCACCTGGTTCAG

Using Gateway cloning (Thermo Fisher Scientific), the *CDK19* cDNA entry clone (GenBank: NM_015076.3) in the pDONR221 vector was shuttled to the pGW-attB-HA^79^. All UAS-cDNA Constructs were inserted into the VK37 (PBac{y[+]-attP}VK00037) docking site by ϕC31 mediated transgenesis^80^.

### Generation of endogenously tagged Cdk8::GFP flies

The Cdk8-superfolder-GFP line (referred to as Cdk8::GFP) was generated as described^43^. Briefly, a fragment with gRNAs targeting 5′UTR (GTTATCGGGAGACAGCTGATTGG) and end of coding region (TTAGAAGGCTAGAAATTAGTAGG) of *cdk8* and 200 bps of homology arms (corresponding to 200 bps upstream of 5’ sgRNA cut site and 200 bps downstream 3’ sgRNA cut site) were synthesized and cloned in pUC57_Kan_gw_OK2 custom vector backbone, generating a homology donor intermediate vector by Genewiz/Azenta. Fragments for the gene coding region (between 5’ sgRNA PAM sequence up to final amino acid before the stop codon), linker-sfGFP-stop codon and Scarless-DsRed were PCR amplified with primers containing overlaps and the fragments were assembled using NEB-HiFi DNA Assembly kit (New England Biolabs #E2621) using manufacturer’s instructions, in the homology donor intermediate linearized by BsaI-HF (NEB #R3535) to generate the homology donor vector for injection. Homology donor vector (250 ng/μL) was injected into *y*^*1*^w^*^; attP40(y + *){nos-Cas9(v* + *)}; iso5* embryos^81^. The resulting G0 males and females were crossed to *y w* flies to screen for the presence of 3XP3-DsRed. The resulting transgenics were PCR-verified with primers that amplify tagged gene-specific amplicons.

### Total ATP determination

For the total ATP determination from the fly heads with indicated genotypes, we followed the protocol from ref. ^82^. For each set, ten heads were taken, and the total ATP was measured using an ATP determination kit (A22066, Life Technologies) as per the manufacturer’s instructions. The ATP levels were normalized with the total protein levels.

### Immunostaining and quantification of mitochondria in adult fly brain

Heads from the adult flies were dissected out in cold PBS, and the immunostaining was performed as per ref. ^83^. Rabbit anti-Drp1 and Mouse anti-ATP5a (Abcam, ab14748) antibodies were used in 1:200 and 1:500 dilutions, respectively. The secondary antibodies, including goat anti-rabbit Alexa488 conjugated and goat anti-mouse Alexa555 conjugated, were used in 1:200 dilution. The images were acquired using a Zeiss880 confocal microscope, and the mitochondrial morphology was analyzed using Mitochondrial Network Analysis (MiNA) plug-in software in ImageJ.

### Transmission electron microscopy

Adult fly retinas were processed for TEM imaging as described^84,85^. Samples were processed using a Ted Pella Bio Wave microwave oven with a vacuum attachment. Adult fly heads were dissected at 25 °C in 4% paraformaldehyde, 2% glutaraldehyde, and 0.1 M sodium cacodylate (pH 7.2). Samples were subsequently fixed at 4 °C for 48 h. 1% osmium tetroxide was used for secondary fixation and subsequently dehydrated in ethanol and propylene oxide, and then embedded in Embed-812 resin (Electron Microscopy Science, Hatfield, PA). 50 nm ultra-thin sections were obtained with a Leica UC7 microtome and collected on Formvar-coated copper grids (Electron Microscopy Science, Hatfield, PA). Specimens were stained with 1% uranyl acetate and 2.5% lead citrate and imaged using a JEOL JEM 1010 transmission electron microscope with an AMT XR-16 mid-mount 16 megapixel CCD camera. For quantification of ultrastructural features, electron micrographs were examined from three different animals per treatment.

### Climbing assays

Flies raised at both 25 and 29 °C were subjected to a climbing assay at 3 days, 1 week, 2 weeks, and 3 weeks after eclosion raised at both 25 and 29 °C. Adult flies were transferred into an empty polystyrene vial with a label at a height of 5 cm. Prior to the assay, flies were gently tapped down to the bottom of the vial, and their climbing in 15 s was recorded and quantified. The test was repeated six times for each vial, and three vials per genotype were tested. When using either muscle or neuronal drivers, defects were only manifested in 2-week-old flies, while ubiquitous knockdown caused climbing defects by day 3.

### Lifespan assay

Adult flies were maintained on standard media at 25 °C and transferred to fresh vials every 3 days. Mortality was scored daily.

### Pupal lethality assay

The numbers of pupal cases were counted 3 days after most control flies had eclosed. Counting includes the number of pupal cases in total and the number of pupal cases with dead pharate adults. The test was repeated three times.

### qRT-PCR

Whole larvae from the third instar larval stage or adult thoraxes were briefly washed in PBS and temporarily stored in RNAlater Stabilization solution (Invitrogen AM7020). Total RNA was extracted using RNeasy Mini Kits (Qiagen 74101). The first strand of cDNA was synthesized using the OneScript Plus cDNA Synthesis Kit (Abm G236). qRT-PCR were performed using SensiFast SYBR Lo-ROX Kit (Bioline 94005) on StepOne Real-time PCR System (Applied Biosystems). Primers used are:

*cdk8* F CATCCGGGTGTTTCTGTCG

*cdk8* R CAGCCCGATGGAACTTAATGAT

*cycC* F AGTTTCCCTACCGCACCAATC

*cycC* R ACAATCAAGCAGCAATCCAGG

*pink1* FAAGCGAGGCTTTCCCCTAC

*pink1* R GCACTACATRGACCACCGATTT

*parkin* F GAAGCCTCCAAGCCTCTAAATG

*parkin* R ACGGACTCTTTCTTCATCGGT

*opa1* F CAAGCTGCGATACATCGTCC

*opa1* R GCAGTCCATCCTTCCATTCC

*marf* F GAGACGACCACCTTTATCAACG

*marf* RCCACCTTCATGTGATCCCG

*drp1* FACAGCCCACTCGATGATCG

*drp1* RAAGCACTTCTTGGTGTGCAG

*rp49* FAGCATACAGGCCCAAGATCG

*rp49* RTGTTGTCGATACCCTTGGGC

### Immunofluorescence staining

Larval body wall muscles and adult thoraxes were dissected in PBS and fixed in 4% paraformaldehyde (PFA) for 15 min at room temperature. Samples were washed with PBS with 0.1% Triton X-100 (PBST). After blocking with 5% BSA in PBST for 1 h at room temperature, samples were incubated with primary antibodies overnight at 4 °C. The following primary antibodies were used: rabbit anti-Drp1 (1:500; Cell Signaling D6C7), Rabbit anti-Drp1 S616 (1:500, Cell Signaling D9A1), mouse anti-HA (1:500; Abm G036), rabbit anti-CDK19 (1:200; Sigma-Aldrich SAB4301196), mouse- anti-Lamin C (1:100, DSHB LC28.26), mouse anti-ATP5α (1:500; Abcam ab14748), and rabbit anti-HA (1:500; Abcam ab9110). After washing with PBST, samples were incubated with Cy3- and/or Alexa Fluor 647-conjugated secondary antibodies (1:500; Jackson ImmunoResearch Laboratories), DAPI (final concentration: 0.2 μg per ml; Invitrogen D1306) for 2 h at room temperature. Samples were mounted in 70% glycerol in PBS after washing. Images were taken on a Nikon Air laser-scanning confocal microscope or a Zeiss LSM880 with an Airyscan confocal microscope and processed using ImageJ.

### Quantification of mitochondrial morphology

Airyscan-processed images of larval body wall muscles were subjected to binary and skeletonize processes using ImageJ prior to morphological quantification. Each processed image was randomly assigned into five sections, and each section was subjected to Mitochondrial Network Analysis (MiNA) to quantify the following three parameters: number of networks (structure with at least one junction), mean length of network branches (mean branch length), and mitochondrial footprint (area). Data collected from five sections with four biological replicates were pooled together for statistics and generating box plots. Airyscan-processed images of adult indirect flight muscles were used in ImageJ for morphological quantification. The length of mitochondria is measured, where number of mitochondria are manually counted. Data collected from two sections with five biological replicates were pooled together for statistics and generating box plots.

### Quantification of muscle fiber width

Airyscan-processed images were randomly assigned into five sections, and muscle fibers within the section were measured using ImageJ. Data collected from five sections were pooled together for statistics and generating box plots.

### ROS staining

ROS assays were performed according to the previously described protocol for in vivo detection of ROS^86^. Briefly, adult thoraxes were dissected in PBS and incubated with 30 µM DHE (Cayman 12013) for 7 min in the dark at room temperature. After rinsing with PBS briefly, samples were fixed in 8% PFA and washed with PBS, each for 5 min before mounting. Images were taken within 2 days.

For the detection of ROS in adult brains, a previously described protocol was used with minor modifications^87^. Day 5 fly brains were dissected in Schneider’s medium (SDM). After brief washing with SDM, the brains were incubated for 5 min with 150 μL DHE (final concentration 30 μM, Invitrogen, Cat# D11347) to detect ROS. After washing (5 min × 3 times) in SDM, brains were mounted with VECTASHIELD Antifade Mounting Medium and then observed immediately with a Leica SP8X confocal microscope. Images were obtained as Z series with the same interval (4 μm) for the whole brain. Z series images were merged by ImageJ (Image-Stacks-Z projection-SUM slices), and then the fluorescence intensity was measured.

### Electroretinogram

ERG recordings were performed as described in refs. ^88,89^. In brief, flies were glued to a slide with Elmer’s Glue. A recording electrode filled with 100 mM NaCl was placed on the eye, and a reference electrode was placed on the fly head. During the recording, a 1 s pulse of light stimulation was given, and the ERG traces of ten flies for each genotype were recorded and analyzed with WinWCP v.5.3.3 software.

### Western blotting

Cells or tissues were lysed with 1× Cell Lysis Buffer (Cell Signaling Technology), supplemented with 1× Protease Inhibitors (Roche), and 1 mM phenylmethylsulfonyl fluoride. Protein lysates with 1× SDS sample buffer were resolved on SDS/PAGE and then transferred to nitrocellulose membranes. Membranes were blocked with 5% skimmed milk or 5% bovine serum albumin. The following primary antibodies were used: rabbit anti-Drp1 (1:2500; Cell Signaling D6C7), rabbit anti-pDrp1S616 (1:2500; Cell Signaling 4494), mouse anti-Myc (1:2500; Millipore clone 4A6), rat anti-HA-peroxidase (1:5000; Sigma-Aldrich clone 3F10), mouse anti-Actin (1:5000; Abcam ab3280), rabbit anti-GFP (1:5000, Thermo Fisher A11122), and mouse anti-HSP60 (1:1000; Thermo Fisher MA3-012). HRP (Jackson ImmunoResearch) conjugated secondary antibodies were used. Images were acquired by Amersham Imager 600.

### Co-immunoprecipitation

Protein extracts were incubated with indicated IgG (Abcam ab104155), HA (Abcam ab214758), or Myc-agarose beads (Abcam ab1253) overnight at 4 °C. The beads were washed with lysis buffer and boiled in 4x Laemmli buffer. The supernatants were then analyzed by Western blotting. For detecting the endogenous *Cdk8::GFP*, protein extracts prepped from adult thoraxes were pre-cleared with IgG (Santa Cruz, sc-2343) for 1 h at 4 °C, lysates were then incubated with either IgG (Santa Cruz, sc-2343) or anti-HA (Santa Cruz, sc-7392) overnight at 4 °C. The beads were washed with lysis buffer and boiled in 4x Laemmli buffer. The supernatants were then analyzed by Western blotting.

### Subcellular fractionation

All preparations were performed on ice. Subcellular fractionation was performed as described in refs. ^90,91^ with slight modification. S2R+ insect cells co-transfected with Cdk8 and Drp1 plasmids were resuspended and homogenized in 0.1% Triton in 200 μL 1x PBS (pH 7.4). About 30 μL of the homogenized solution was collected as the whole lysate. The rest of the solution was centrifuged at 10,000 × *g* for 30 s. About 30 μL of supernatant was collected as cytoplasmic fraction. To clear out the debris in the nuclear fraction, five subsequent washings with 0.1% Triton were performed, followed by centrifugation at 10000×*g* for 1 min per washing. Whole lysate and cytoplasmic fractions were boiled for 5 min with 4x Laemmli, whereas the nuclear fractions were boiled for 10 min with 1x Laemmli, and sonicated with 2 pulse, 5 s each.

### Kinase assays

Clean cytoplasmic fractions from S2R+ insect cells containing pAWM-Cdk8 and pAWH-Drp1 from subcellular fractionations were subjected to co-immunoprecipitation with Myc-agarose beads (Abcam ab1253) overnight at 4 °C. The beads were washed with lysis buffer and centrifugated at 3000 × *g* for 2 min at 4 °C. Beads were kept to perform subsequent kinase assay following protocol listed in ref. ^92^ with a final concentration of 0.2 mM ATP added. The reactions were paused after 30 min, centrifugated at max speed for 5 min at 4 °C, and boiled for 10 min with 4x Laemmli. The level of Cdk8, Drp1, and pDrp1 were detected using mouse anti-Myc (1:2500; Millipore clone 4A6), rabbit anti-Drp1 (1:2500; Cell Signaling D6C7), rabbit anti-pDrp1S616 (1:2500; Cell Signaling 4494) on western blots, respectively.

### Plasmids and cell culture

Entry clones were created from ORF by TOPO cloning (pENTR-D-TOPO, Thermo Fisher) according to the manufacturer’s instruction. The ORFs for *Cdk8* [CG10572, FMO10168 (DGRC Stock 1637668; https://dgrc.bio.indiana.edu//stock/1637668; RRID:DGRC_1637668)] and *Drp1* [CG3210, FI19305 (DGRC Stock 1647302; https://dgrc.bio.indiana.edu//stock/1647302; RRID:DGRC_1647302)] were from the DGRC clone collection. All ORFs were amplified by PCR using primers bearing flanking attB1 and attB2 sequences and subjected to BP recombination reactions using the pDONR/Zeo vector (Invitrogen) to generate Entry vectors. Expression vectors were obtained through LR recombination reactions using the pAWM and pAWH vectors from the DGRC Gateway collection.

Drosophila S2R+ cells (DGRC stock 150) were maintained at 25 °C in Schneider’s medium supplemented with 10% heat-inactivated FCS (Thermo Fisher Scientific). Cells were transfected with Effectene (Qiagen) according to the manufacturer’s recommendation. Twenty-four hours after transfection, cells were lysed for subsequent biochemical analysis.

### Statistical analyses

We used GraphPad Prism for statistical analysis and generation of figures. Statistical analysis was done with the default settings of the software (∗ indicates *p* < 0.05, ∗∗ indicates *p* < 0.01, ∗∗∗ indicates *p* < 0.001, ∗∗∗∗ indicates *p* < 0.0001). Violin plots were generated using BoxPlotR (Spitzer et al. 2014) to demonstrate the average distribution of climbing assays.

### Web resources

cNLS Mapper: https://nls-mapper.iab.keio.ac.jp/cgi-bin/NLS_Mapper_form.cgi.

### Reporting summary

Further information on research design is available in the Nature Portfolio Reporting Summary linked to this article.

### Supplementary information

Supplementary Information

Reporting Summary

### Source data

Source data

## Data Availability

Source data are provided with this paper.
