# Supplementary material for: Cdk8/CDK19 promotes mitochondrial fission through Drp1 phosphorylation and can phenotypically suppress pink1 deficiency in Drosophila
Source: Nat Commun. 2024 Apr 18;15:3326. doi: 10.1038/s41467-024-47623-8 (PMC11026413; doi:10.1038/s41467-024-47623-8)
Supplement: Supplementary file 1 — Supplementary Information [file 41467_2024_47623_MOESM1_ESM.pdf]

Figure S1. Depletion of Cdk8 causes behavioral defects.

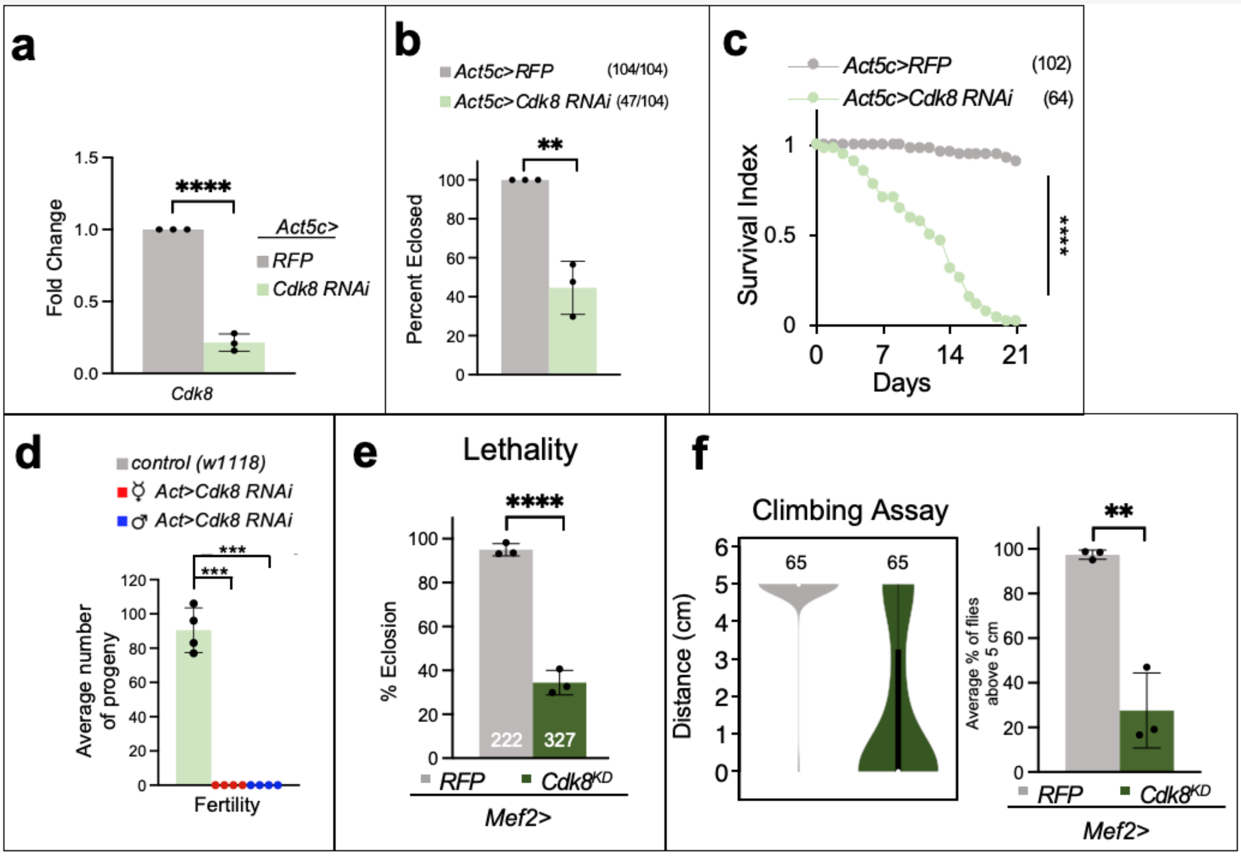

(a) qRT-PCR analysis of *Cdk8* gene expression in *Act5c>Cdk8 RNAi* relative to control sample (*Act5c>RFP*). Data are mean ± SD. *n* = 3 independent experiments. **(b)** Semi-lethality is found in *Act5c>Cdk8 RNAi* pupae in comparison to *Act5c>RFP* (n=104 per genotype). Data are mean ± SD. **(c)** Life span assay between *Act5c>RFP* and *Act5c>Cdk8 RNAi*. **(d)** Infertility is found in both female and male *Act5c> Cdk8 RNAi* flies when crossed to w^{1118} in comparison to control (w^{1118}). Data are mean ± SD. *n* = 4 independent experiments **(e)** Semi-lethality is found in Mef2>Cdk8KD pupae compared to Mef2>RFP. Data are mean ± SD. **(f)** Violin plots showing distributions of climbing ability in control (*Mef2>RFP*) and in *Mef2>Cdk8*^{KD}. Average percent of flies that reached the target line of indicated genotypes. Data are mean ± SD All raised at 25°C. All statistics are generated using unpaired two-tailed t-test. ** p<0.01, *** p<0.001, **** p<0.0001, ns: no significance. Source data are provided as a Source Data file.

Figure S2. Cdk8 and Cyclin C do not affect *pink1* or *parkin* transcription and do not interact to regulate mitochondrial dynamics

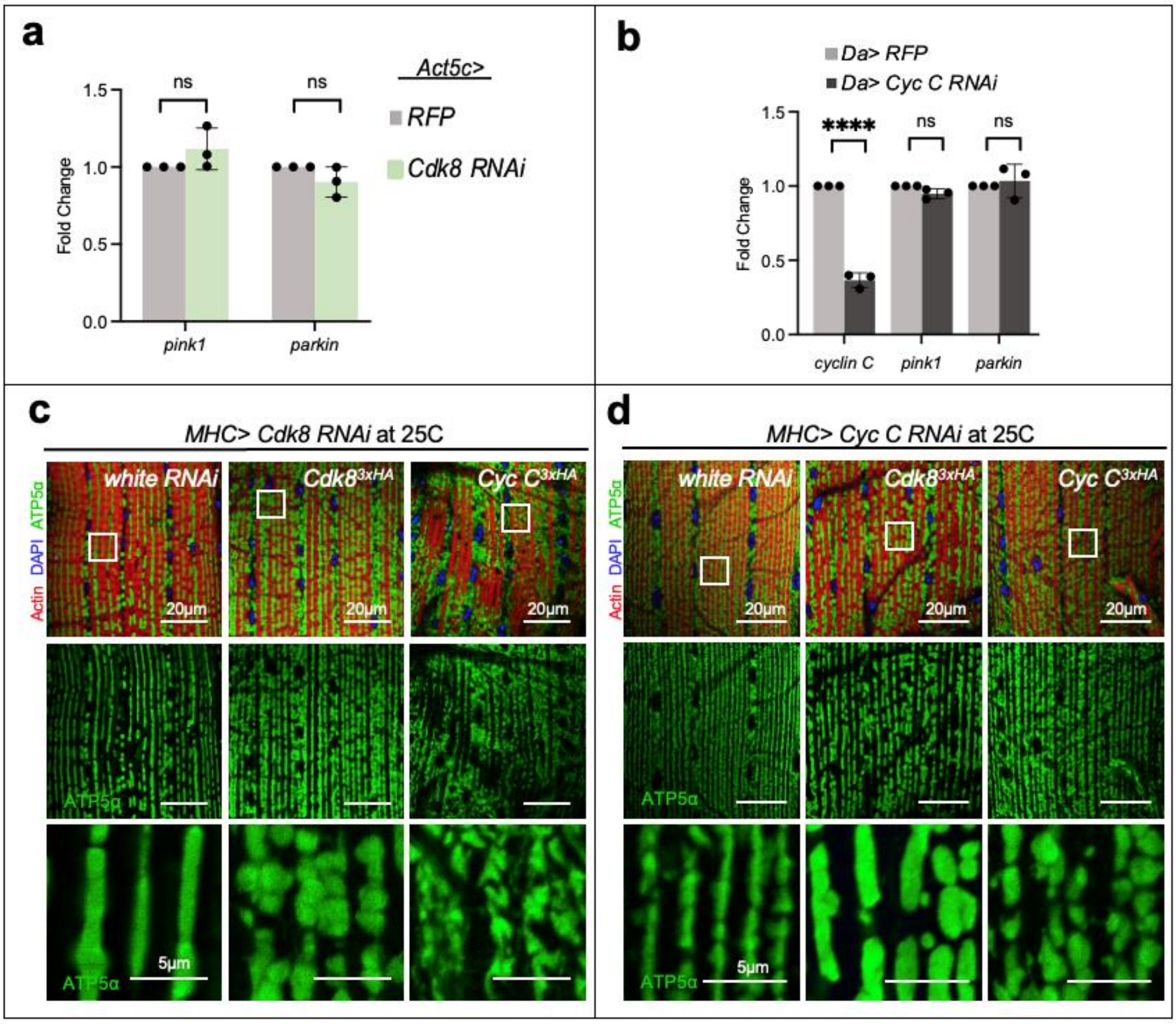

(a) qRT-PCR analysis of pink1 and parkin gene expression in Act5c>Cdk8 RNAi relative to control sample (Act5c>RFP). Data are mean ± SD. n = 3 independent experiments. Unpaired two-tailed t-test is used to generate the statistics. **(b)** qPCR analysis of *CycC*, *pink1* and *parkin* gene expression following *CycC* RNAi expression relative to control (*da>RFP*). Data are mean ± SD. n = 3 independent experiments. Unpaired two-tailed t-test is used to generate the statistics. **(c)** Mitochondrial morphology of adult IFM expressing either *white RNAi*, *Cdk8^{HA}* or *CycC^{HA}* in a *Cdk8* depleted background. Scale bar: 20 µm. A representative magnified section with corresponding genotypes is included. Scale bar: 5 µm. Raised at 25°C. (n = 6 per genotype) **(d)** Mitochondrial morphology of adult IFM expressing either *white RNAi* , *Cdk8^{HA}* or *Cyc C^{HA}* in a *Cyc C* depleted background. Scale bar: 20 µm. A representative magnified section with corresponding genotypes is included. Scale bar: 5 µm. Raised at 25°C. (n = 7 per genotype) **** p<0.0001, ns: no significance. Source data are provided as a Source Data file.

Figure S3. Cdk8/CDK19 and CycC regulate mitochondrial morphology under physiological conditions during development and adult homeostasis.

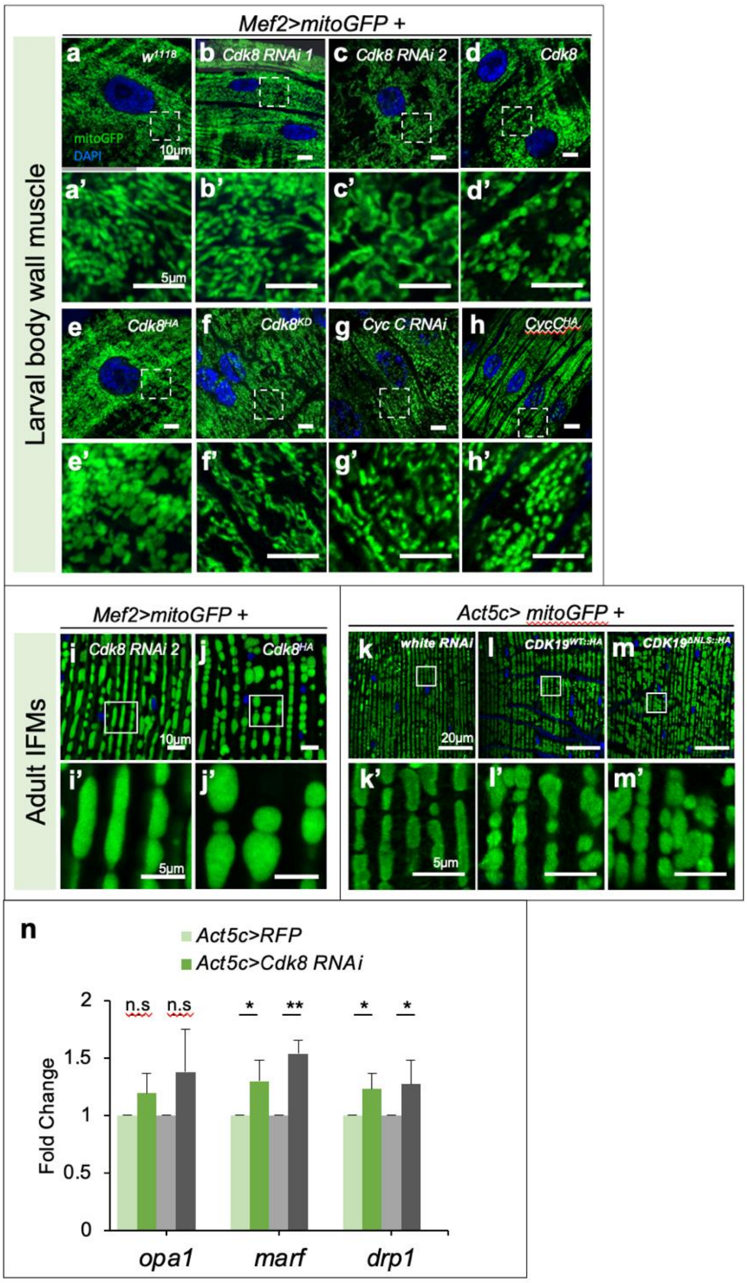

Mitochondrial morphology of larval body wall muscles in **(a)** control or expressing **(b)** *Cdk8 RNAi 1*, **(c)** *Cdk8 RNAi 2*, **(d)**$Cdk8^{WT}$, **(e)** $Cdk8^{HA}$, **(f)** $Cdk8^{KD}$, **(g)** *CycC RNAi* or **(h)** $CycC^{HA}$. Scale bar: 10 µm. **(a'-h')** Representative magnified sections from a-h (indicated with dashed boxes). Scale bar: 5 µm. **b-i** raised at 25°C. **(i-j)** Mitochondrial morphology of adult IFM expressing **(i)** *Cdk8 RNAi 2*, **(j)** $Cdk8^{HA}$. Scale bar: 10 µm. **(i'-j')** A representative magnified section from **(i-j)** with corresponding genotypes listed above. Scale bar: 5 µm. Raised at 29°C. **(k-m)** Mitochondrial morphology of adult IFM expressing **(k)** *Act5c>white RNAi* (control), **(l)** *Act5c>CDK19WT*, **(m)** *Act5c>*$CDK19^{\Delta NLS}$. Scale bar: 20 µm. **(k'-m')** A

representative magnified section from **(k-m)** with corresponding genotypes listed above. Scale bar: 5 µm. **(n)** qPCR analysis of mitochondrial dynamic regulator gene expressions in both *Act5c>Cdk8* RNAi and *Da>CycC* RNAi relative to controls (*Act5c>RFP* or *Da>RFP*). Data are mean ± SD. Unpaired two-tailed t-test is used to generate the statistics. n = 3 independent experiments. Source data are provided as a Source Data file.

Figure S4. Depletion of Cdk8 caused elevated reactive oxygen species (ROS) which can be suppressed by CDK19 expression

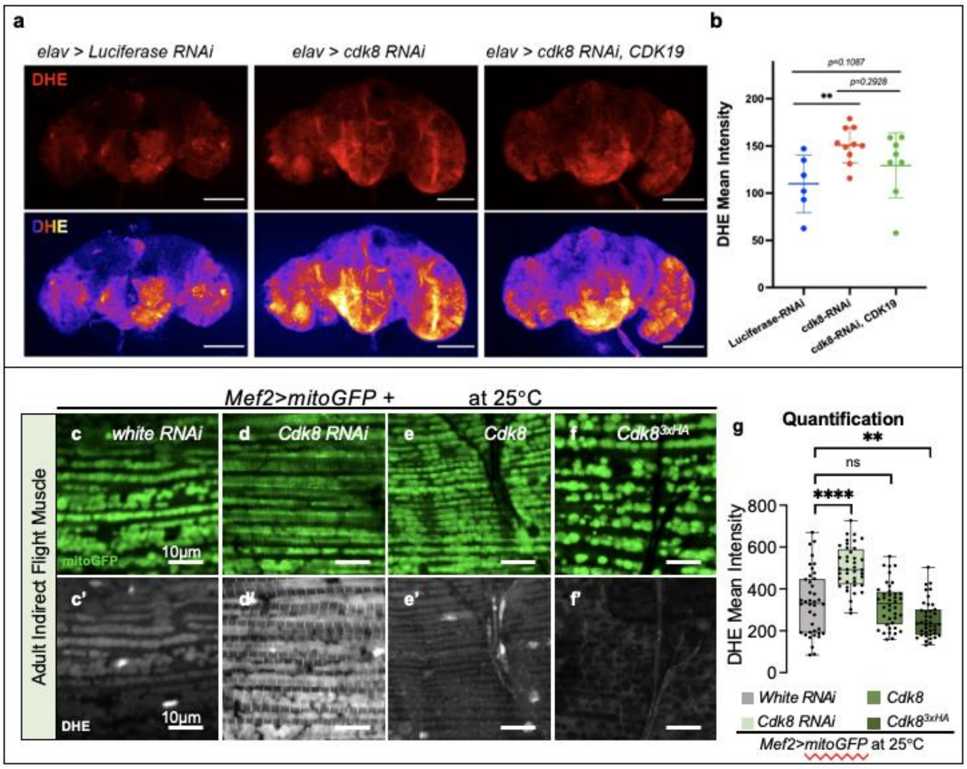

(a) DHE staining in adult brains of the indicated genotypes. **(b)** Quantification of DHE mean intensity in brains shown in **(a)**. **(c-f)** Mitochondrial morphology of adult IFM of the indicated genotypes where transgenes are expressed in muscles using *Mef2-Gal4* and mitochondria are detected using *UAS-mitoGFP*. Scale bar: 10 µm. **(c'-f')** DHE staining of the indicated genotypes. **(g)** Quantification of DHE mean intensity for panels c'-f'. n = 8 per genotype. Data are presented as box plots (centre line at the median, upper bound at 75th percentile, lower bound at 25th percentile) with whiskers at minimum and maximum values. Unpaired two tailed t-test is used to generate the statistics. **p < 0.01, ****p < 0.0001, ns: no significance. Source data are provided as a Source Data file.

Figure S5. Identifying Drp1 as a potential target of Cdk8

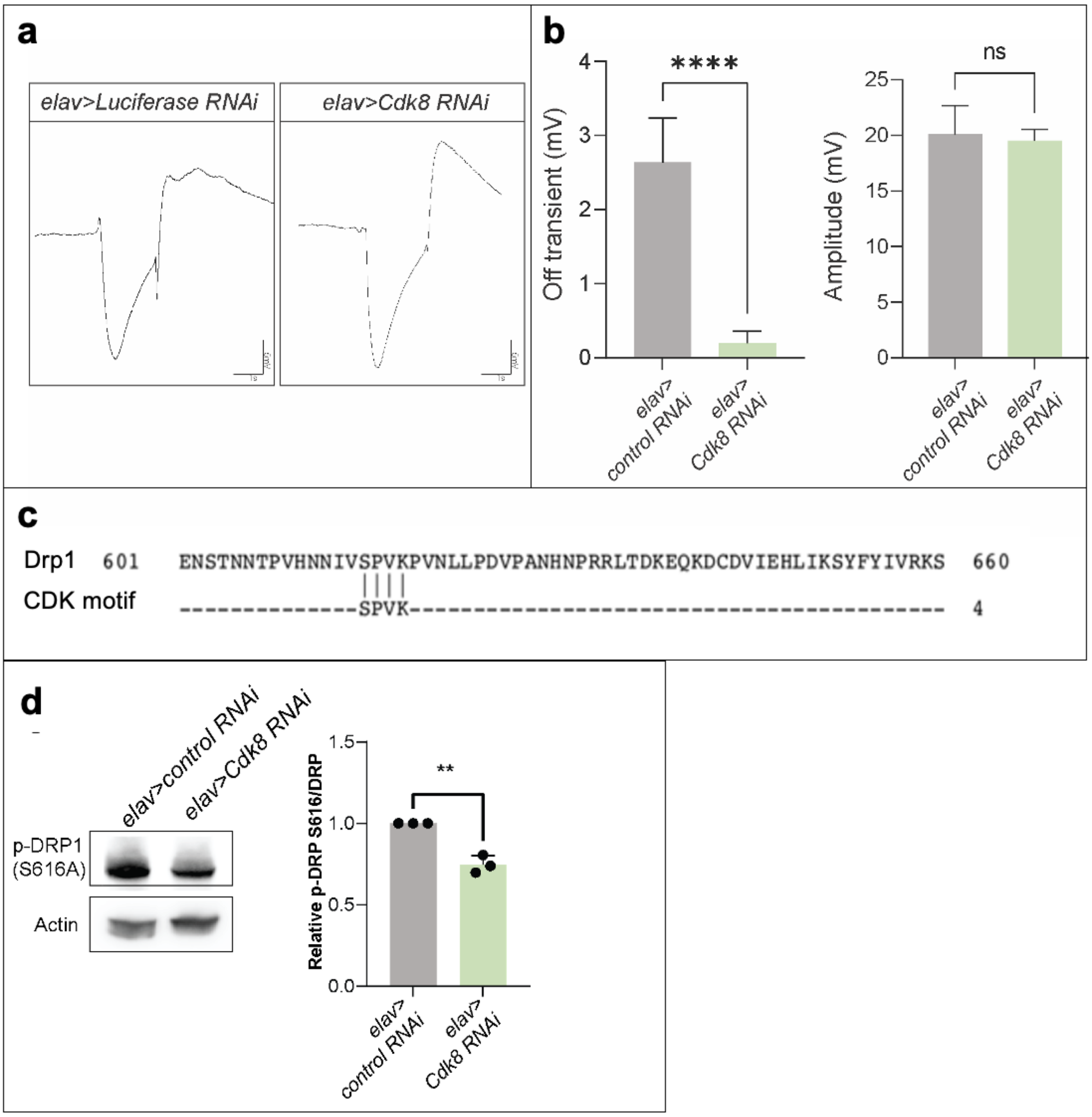

(a) ERG traces of control (*elav>luciferase RNAi*) and the flies that reduce the level of Cdk8 (*elav>Cdk8 RNAi*). **(b)** Quantification of off transient (left) and depolarization amplitude (right). **(c)** Protein sequence alignment between the fission regulator Drp1 and the consensus CDK phosphorylation motif. **(d)** Western blot results showing the level of phosphor Drp1 S616 and its quantification versus total DRP1 level in the heads of control (*elav>control RNAi*), and flies that express *Cdk8 RNAi* (*elav>Cdk8 RNAi*) (n=3 per genotype). *p < 0.05, **p < 0.01, ****p < 0.0001, ns: no significance. Source data are provided as a Source Data file.

Figure S6. Sequence alignment between fly Cdk8 and human CDK19 with predicted location for nuclear localization sequence.

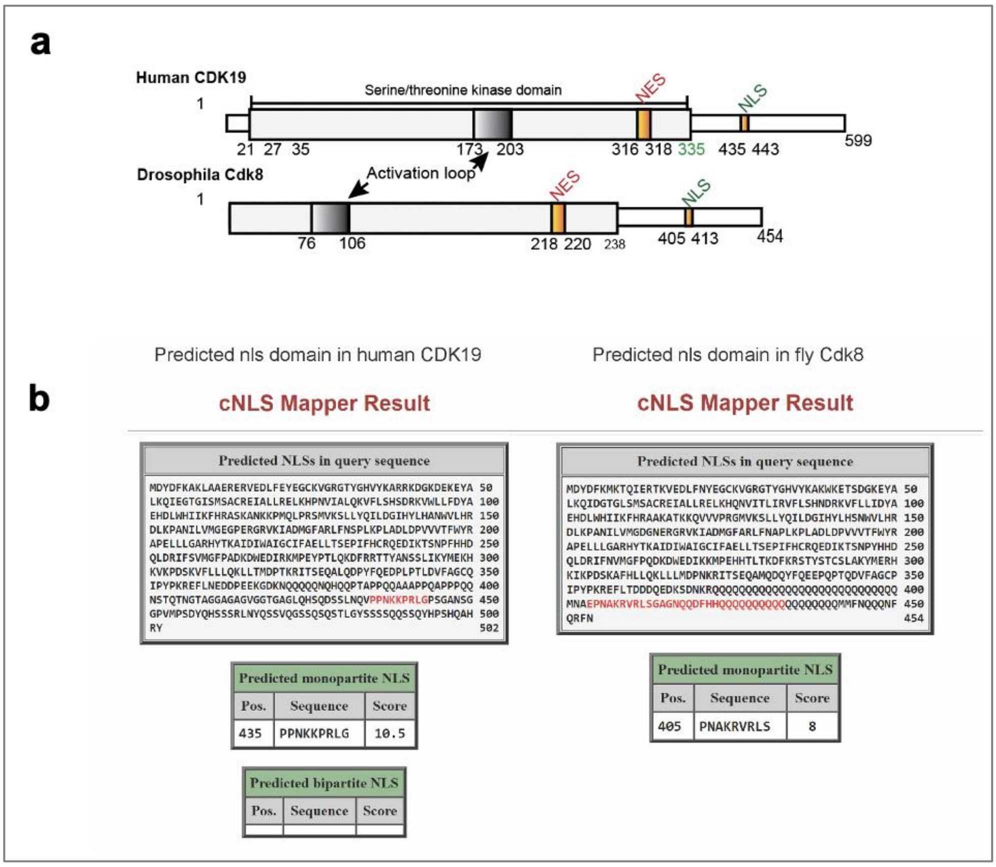

(a) Schematic image showing the key domains of human CDK19 and fly Cdk8, including NES and NLS. **(b)** NLS domain prediction of human CDK19 (435-443) and fly Cdk8 (405-413) using cNLS Mapper (https://nls-mapper.iab.keio.ac.jp/cgi-bin/NLS_Mapper_form.cgi). This program is based on Kosugi et al., (2009) PNAS 106, 10171-10176.

Figure S7. Localization of endogenously tagged Cdk8 with its potential interaction with Drp1

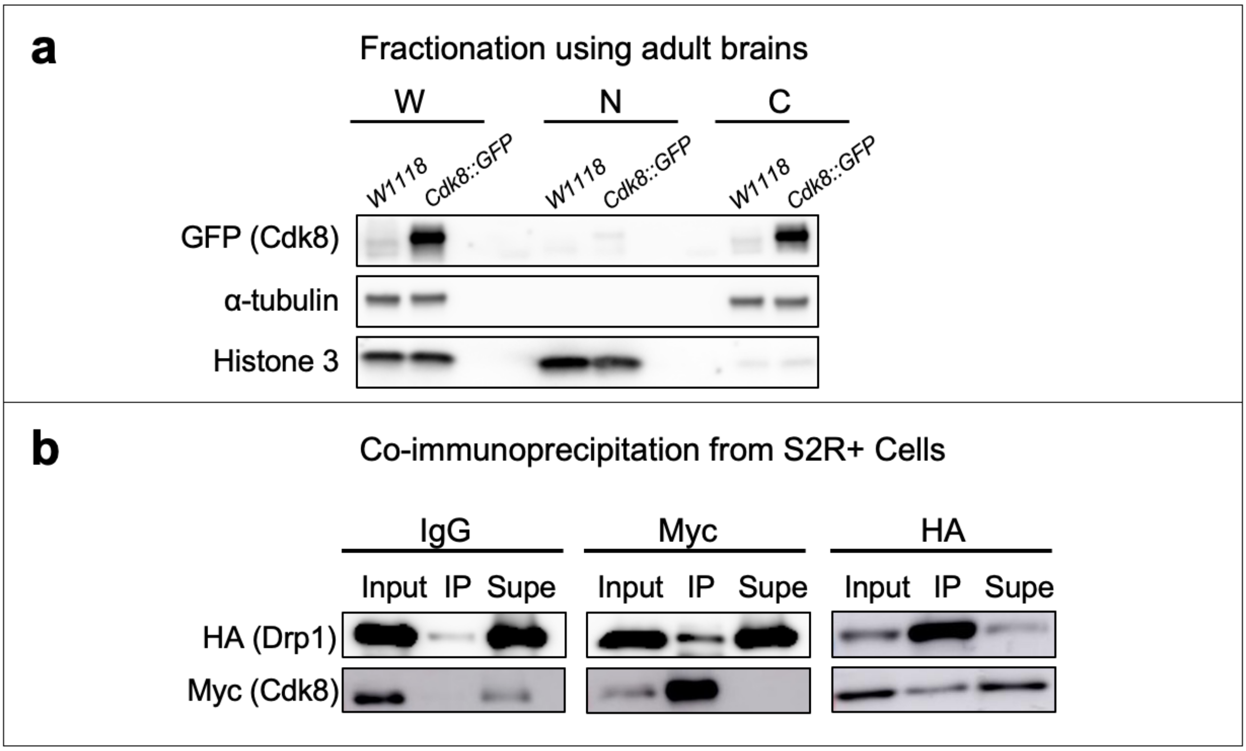

(a) Fractionation from adult brains from *w*^{1118} or *Cdk8::GFP* flies. Whole cell lysate (W), nuclear (N) and cytoplasmic (C) fractions were prepared, and antibodies to a-tubulin and Histone 3 were used to detect the cytoplasmic and nuclear fractions, respectively. **(b)** Co-immunoprecipitation using S2R+ cells co-transfected with HA-tagged Drp1 and Myc-tagged Cdk8 plasmids. IgG agarose beads were used as negative control. IPs used HA-agarose beads, or the reciprocal pulldown with Myc-agarose beads. Source data are provided as a Source Data file.

Figure S8. Ectopic Cdk8 expression can rescue defects found in aged *pink1^{B9}* mutants.

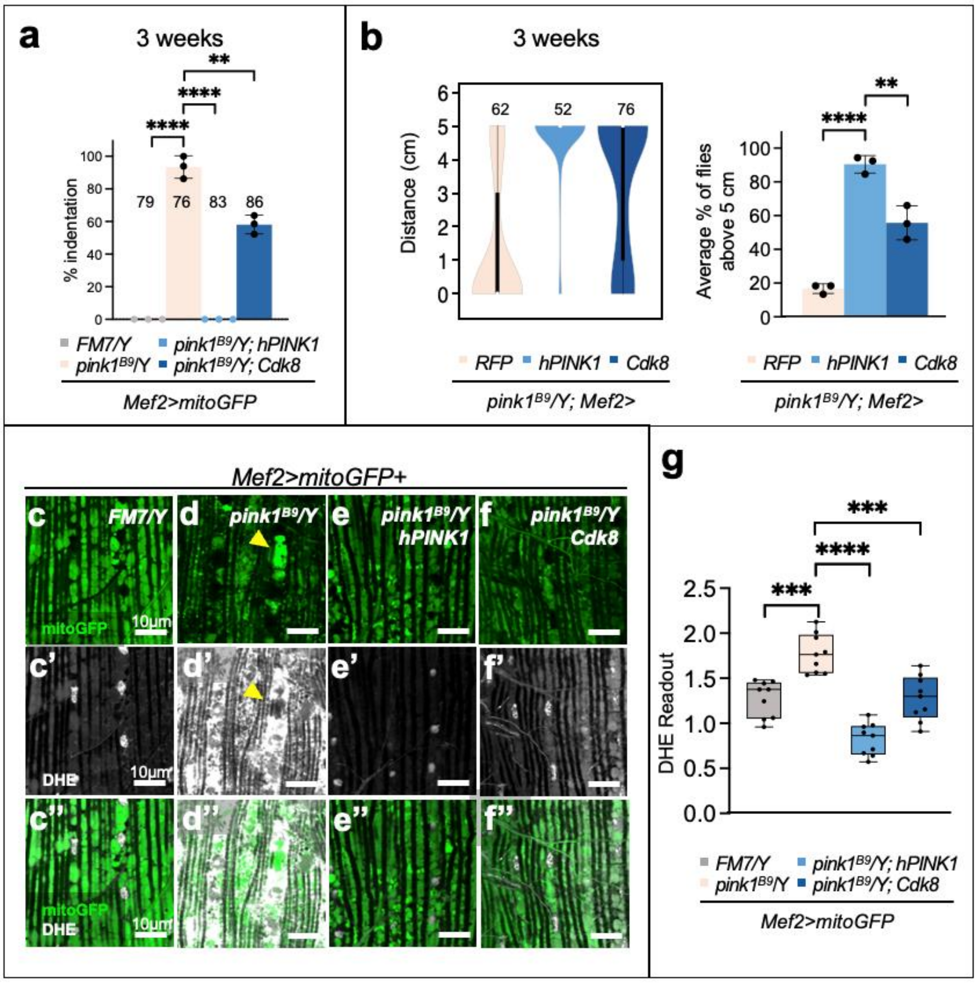

(a) Quantification of the thorax indentation in three weeks old flies expressing either RFP, hPink1 or Cdk8 in a *pink1^{B9}* mutant background. Total number of progeny are assessed, n = 79, 76, 83, 86 respectively in order. Results are mean ± SD. Unpaired two tailed t-test is used to generate the statistics. **(b)** Left - Violin plots showing distribution of climbing activity of three weeks old flies expressing RFP, hPink1 or Cdk8 in a *pink1^{B9}* mutant background. Right- Average percent of flies that reached the target line. Results are mean ± SD. Unpaired two tailed t-test is used to generate the statistics. **(c-f)** IFM in **(c)** *FM7/Y*, **(d)** *pink1^{B9}*/Y, and **(e)** *hPink1* or **(f)** *Cdk8* both expressed in the *pink1^{B9}* mutant background with *Mef2>mitoGFP*. **(c'-f')** DHE staining of genotypes indicated in **(c-f)**. **(c''-f'')** Merged images. Scale bar: 10 µm. **(g)** Quantification of the DHE fluorescent intensity using spectrophotometry. Data are presented as box plots (centre line at the median, upper bound at 75th percentile, lower bound at 25th percentile) with whiskers at minimum and maximum values. Unpaired two tailed t-test is used to generate the statistics. n = 3 independent experiments. All raised at 29°C. *p < 0.05, ** p<0.01, *** p<0.001, **** p<0.0001, ns: no significance. Source data are provided as a Source Data file.
