# Supplementary material for: Cdk8/CDK19 promotes mitochondrial fission through Drp1 phosphorylation and can phenotypically suppress pink1 deficiency in Drosophila
Source: Nat Commun. 2024 Apr 18;15:3326. doi: 10.1038/s41467-024-47623-8 (PMC11026413; doi:10.1038/s41467-024-47623-8)
Supplement: Supplementary file 2 — Reporting Summary [file 41467_2024_47623_MOESM2_ESM.pdf]

nature portfolio

Corresponding author(s): Bellen and Verheyen

Last updated by author(s): March 18, 2024

Reporting Summary

Nature Portfolio wishes to improve the reproducibility of the work that we publish. This form provides structure for consistency and transparency in reporting. For further information on Nature Portfolio policies, see our Editorial Policies and the Editorial Policy Checklist.

Statistics

For all statistical analyses, confirm that the following items are present in the figure legend, table legend, main text, or Methods section.

n/a	Confirmed	
☐	☒	The exact sample size (*n*) for each experimental group/condition, given as a discrete number and unit of measurement
☐	☒	A statement on whether measurements were taken from distinct samples or whether the same sample was measured repeatedly
☐	☒	The statistical test(s) used AND whether they are one- or two-sided<br>*Only common tests should be described solely by name; describe more complex techniques in the Methods section.*
☒	☐	A description of all covariates tested
☒	☐	A description of any assumptions or corrections, such as tests of normality and adjustment for multiple comparisons
☐	☒	A full description of the statistical parameters including central tendency (e.g. means) or other basic estimates (e.g. regression coefficient) AND variation (e.g. standard deviation) or associated estimates of uncertainty (e.g. confidence intervals)
☐	☒	For null hypothesis testing, the test statistic (e.g. *F*, *t*, *r*) with confidence intervals, effect sizes, degrees of freedom and *P* value noted<br>*Give P values as exact values whenever suitable.*
☒	☐	For Bayesian analysis, information on the choice of priors and Markov chain Monte Carlo settings
☒	☐	For hierarchical and complex designs, identification of the appropriate level for tests and full reporting of outcomes
☒	☐	Estimates of effect sizes (e.g. Cohen's *d*, Pearson's *r*), indicating how they were calculated

Our web collection on statistics for biologists contains articles on many of the points above.

Software and code

Policy information about availability of computer code

Data collection	No software was used for data collection.
Data analysis	Mitochondrial Network Analysis (MiNA), ImageJ plug-in version MiNA2.0.0; GraphPad Prism9.1.0.

For manuscripts utilizing custom algorithms or software that are central to the research but not yet described in published literature, software must be made available to editors and reviewers. We strongly encourage code deposition in a community repository (e.g. GitHub). See the Nature Portfolio guidelines for submitting code & software for further information.

Data

Policy information about availability of data

All manuscripts must include a data availability statement. This statement should provide the following information, where applicable:

- Accession codes, unique identifiers, or web links for publicly available datasets
- A description of any restrictions on data availability
- For clinical datasets or third party data, please ensure that the statement adheres to our policy

All data supporting the findings of this study are available within the paper and its Supplementary Information.

Research involving human participants, their data, or biological material

Policy information about studies with [human participants or human data](). See also policy information about [sex, gender (identity/presentation), and sexual orientation]() and [race, ethnicity and racism]().

Reporting on sex and gender	N/A
Reporting on race, ethnicity, or other socially relevant groupings	N/A
Population characteristics	N/A
Recruitment	N/A
Ethics oversight	N/A

Note that full information on the approval of the study protocol must also be provided in the manuscript.

Field-specific reporting

Please select the one below that is the best fit for your research. If you are not sure, read the appropriate sections before making your selection.

☒ Life sciences ☐ Behavioural & social sciences ☐ Ecological, evolutionary & environmental sciences

For a reference copy of the document with all sections, see [nature.com/documents/nr-reporting-summary-flat.pdf]()

Life sciences study design

All studies must disclose on these points even when the disclosure is negative.

Sample size	For each experiment, sample sizes varied slightly based on sample availability. Overall values conformed to norms in the field. In brief, we evaluated between 3-10 samples per genotype, per biological replicate, as indicated in figure legends.
Data exclusions	No data were excluded from the study.
Replication	All experiments consisted of three biological replicates and all attempts were successful.
Randomization	No sample randomization was performed because the samples are defined by unique genotypes. However, within the genotypes, the animals were allocated randomly.
Blinding	Blinding was not necessary as all data sets were quantified to eliminate bias.

Reporting for specific materials, systems and methods

We require information from authors about some types of materials, experimental systems and methods used in many studies. Here, indicate whether each material, system or method listed is relevant to your study. If you are not sure if a list item applies to your research, read the appropriate section before selecting a response.

Materials & experimental systems

n/a	Involved in the study	
☐	☒	Antibodies
☒	☐	Eukaryotic cell lines
☒	☐	Palaeontology and archaeology
☐	☒	Animals and other organisms
☒	☐	Clinical data
☒	☐	Dual use research of concern
☒	☐	Plants

Methods

n/a	Involved in the study	
☒	☐	ChIP-seq
☒	☐	Flow cytometry
☒	☐	MRI-based neuroimaging

Antibodies

Antibodies used	rabbit anti-Drp1 (1:500; Cell Signaling D6C7), mouse anti-HA (1:500; Abm G036), mouse anti-ATP5α (1:500; Abcam ab14748), rabbit anti-HA (1:500; Abcam ab9110), rabbit anti-pDrp1S616 (1:2500; Cell Signaling 4494), mouse anti-Myc (1:2500; Millipore clone 4A6), rat anti-HA-peroxidase (1:5000; Sigma Aldrich clone 3F10), mouse anti-Actin (1:5000; Abcam ab3280), mouse anti-HSP60 (1:1000;

	ThermoFisher MA3-012), rabbit anti-CDK19 (1:200; Sigma-aldrich SAB4301196), mouse- anti-Lamin C (1:100, DSHB LC28.26), rabbit anti-GFP (1:5000, ThermoFisher A11122), mouse anti-α tubulin (1:5000; DSHB AA4.3)<br>rabbit anti-Histone 3 (1:5000; Cell Signaling 9715).
Validation	Validation and/or use of antibodies in the listed publications:<br>rabbit anti-Drp1 (1:5000; Cell Signaling D6C7 #8570) and rabbit anti-pDrp1S616 (1:2500; Cell Signaling 4494)<br>PMID: 32484300<br><br>mouse anti-HA (1:500; Abm G036),<br>PMID: 22048023<br><br>rabbit anti-HA (1:500; Abcam ab9110),<br>PMID: 26429885<br><br>mouse anti-ATP5α (1:500; Abcam ab14748),<br>PMID: 32444642<br><br>mouse anti-Myc (1:2500; Millipore clone 4A6),<br>PMID: 22733779<br><br>rat anti-HA-peroxidase (1:5000; Sigma Aldrich clone 3F10),<br>PMID: 25651184<br><br>mouse anti-Actin (1:5000; Abcam ab3280),<br>PMID: 26153232<br><br>mouse anti-HSP60 (1:1000; ThermoFisher MA3-012),<br>PMID: 36690850<br><br>rabbit anti-CDK19 (1:200; Sigma-aldrich SAB4301196),<br>PMID: 32330417<br><br>mouse- anti-Lamin C (1:100, DSHB LC28.26),<br>PMID: 31300663<br><br>rabbit anti-GFP (1:5000, ThermoFisher A11122)<br>PMID: 37735159<br><br>mouse anti-α tubulin (1:5000; DSHB AA4.3)<br>PMID: 33026342<br><br>rabbit anti-Histone 3 (1:5000; Cell Signaling 9715)<br>PMID: 24788460

Animals and other research organisms

Policy information about studies involving animals; ARRIVE guidelines recommended for reporting animal research, and Sex and Gender in Research

Laboratory animals	Drosophila melanogaster, aged between 1-30 days. Exact genotypes are indicated in Methods.
Wild animals	No wild animals were used in this study.
Reporting on sex	In climbing assays, male and female flies were assessed separately. In interaction studies with pink1 mutant flies, only males were examined since the mutation maps to the X-chromsome.
Field-collected samples	No filed-collected samples were used in this study.
Ethics oversight	No ethical approval was required for these studies, as Drosophila are exempt from ethics rules.

Note that full information on the approval of the study protocol must also be provided in the manuscript.
